# Computational modelling in disorders of consciousness: Closing the gap towards personalised models for restoring consciousness

**DOI:** 10.1016/j.neuroimage.2023.120162

**Published:** 2023-07-15

**Authors:** Andrea I. Luppi, Joana Cabral, Rodrigo Cofre, Pedro A.M. Mediano, Fernando E. Rosas, Abid Y. Qureshi, Amy Kuceyeski, Enzo Tagliazucchi, Federico Raimondo, Gustavo Deco, James M. Shine, Morten L. Kringelbach, Patricio Orio, ShiNung Ching, Yonatan Sanz Perl, Michael N. Diringer, Robert D. Stevens, Jacobo Diego Sitt

**Affiliations:** a Division of Anaesthesia and Department of Clinical Neurosciences, University of Cambridge, Cambridge, UK; b Montreal Neurological Institute, McGill University, Montreal, Quebec, Canada; c Life and Health Sciences Research Institute, University of Minho, Portugal; d CIMFAV-Ingemat, Facultad de Ingeniería, Universidad de Valparaíso, Valparaíso, Chile; e Centre National de la Recherche Scientifique (CNRS), Institute of Neuroscience (NeuroPSI), Paris-Saclay University, Gif-sur-Yvette, France; f Department of Computing, Imperial College London, London, UK; g Department of Psychology, University of Cambridge, Cambridge, UK; h Department of Informatics, University of Sussex, Brighton, UK; i Centre for Psychedelic Research, Department of Brain Sciences, Imperial College London, London, UK; j Centre for Complexity Science, Imperial College London, London, UK; k Centre for Eudaimonia and Human Flourishing, Linacre College, University of Oxford, Oxford, UK; l University of Kansas Medical Center, Kansas City, MO, USA; m Department of Radiology, Weill Cornell Medicine, New York, USA; n Departamento de Física (UBA) e Instituto de Fisica de Buenos Aires (CONICET), Buenos Aires, Argentina; o Latin American Brain Health Institute (BrainLat), Universidad Adolfo Ibáñez, Santiago, Chile; p Institute of Neuroscience and Medicine (INM-7: Brain and Behaviour), Research Centre Jülich, Germany; q Institute of Systems Neuroscience, Heinrich Heine University Düsseldorf, Düsseldorf, Germany; r Center for Brain and Cognition, Department of Information and Communication Technologies, Universitat Pompeu Fabra, Barcelona, Spain; s Department of Neuropsychology, Max Planck Institute for Human Cognitive and Brain Sciences, Leipzig, Germany; t Institució Catalana de Recerca i Estudis Avançats (ICREA), Barcelona, Spain; u Turner Institute for Brain and Mental Health, Monash University, Melbourne, VIC, Australia; v Brain and Mind Center, The University of Sydney, Sydney, Australia; w Department of Psychiatry, University of Oxford, Oxford, UK; x Center for Music in the Brain, Department of Clinical Medicine, Aarhus University, Aarhus, Denmark; y Centro Interdisciplinario de Neurociencia de Valparaíso and Instituto de Neurociencia, Universidad de Valparaíso, Valparaíso, Chile; z Electrical and Systems Engineering, Washington University in St. Louis, St. Louis, MO, USA; aa Institut du Cerveau et de la Moelle épinière - Paris Brain Institute, ICM, Paris, France; ab National Scientific and Technical Research Council (CONICET), Godoy Cruz, CABA 2290, Argentina; ac Department of Neurology and Neurosurgery, Washington University in St. Louis, St. Louis, MO, USA; ad Departments of Anesthesiology and Critical Care Medicine, Neurology, and Biomedical Engineering, Johns Hopkins University, Baltimore, MD, USA; ae Sorbonne Université, Inserm, CNRS, APHP, Hôpital de la Pitié Salpêtrière, Paris, France

**Keywords:** Disorders of consciousness, Computational models, Generative models, Statistical models, Biophysical models, Machine learning

## Abstract

Disorders of consciousness are complex conditions characterised by persistent loss of responsiveness due to brain injury. They present diagnostic challenges and limited options for treatment, and highlight the urgent need for a more thorough understanding of how human consciousness arises from coordinated neural activity. The increasing availability of multimodal neuroimaging data has given rise to a wide range of clinically- and scientifically-motivated modelling efforts, seeking to improve data-driven stratification of patients, to identify causal mechanisms for patient pathophysiology and loss of consciousness more broadly, and to develop simulations as a means of testing *in silico* potential treatment avenues to restore consciousness. As a dedicated Working Group of clinicians and neuroscientists of the international Curing Coma Campaign, here we provide our framework and vision to understand the diverse statistical and generative computational modelling approaches that are being employed in this fast-growing field. We identify the gaps that exist between the current state-of-the-art in statistical and biophysical computational modelling in human neuroscience, and the aspirational goal of a mature field of modelling disorders of consciousness; which might drive improved treatments and outcomes in the clinic. Finally, we make several recommendations for how the field as a whole can work together to address these challenges.

## Highlights

• Overview of the wide range of modelling strategies for disorders of consciousness.
• Descriptive and generative statistical models, biophysical computational models.
• Gap analysis of challenges to DOC modelling and recommendations to overcome them.
• Towards personalised models for diagnosis and treatment of DOC with multimodal data.
• “Phase Zero” in silico clinical trials of potential treatments via brain modelling.

## 1 Introduction

### 1.1 Disorders of consciousness

Advances in intensive care medicine have led to an increasing number of patients surviving severe brain injuries. Some of these patients regain consciousness, but others will remain in a Disorder of Consciousness (DOC) state. Post-comatose DOC patients are characterized by having their eyes open (awake) while remaining seemingly unable to respond or communicate (Claassen et al., 2021; Giacino, 2004). Chronic impairments of consciousness constitute a challenging situation, which requires better understanding of the underlying physiopathology to develop novel diagnostic, prognostic, and therapeutic tools to individually and optimally take care of each patient (Edlow et al., 2020). This clinical goal is well aligned (though of course not identical) with the scientific goal of a robust theory of consciousness (Luppi et al., 2021). Improving our ability to detect consciousness and promote its recovery will enhance our ability to study it; and in turn, obtaining a better understanding of consciousness and its neural bases is expected to increase our ability to identify its presence or the causes of its absence, and how to promote its recovery in the clinic.

Patients with disorders of consciousness occupy a behavioural spectrum currently divided into different conditions: coma, vegetative state or unresponsive wakefulness syndrome (VS/UWS) and minimally conscious state (MCS). Patients in a coma are characterised by lack of arousal and responsiveness. On the other hand, both VS/UWS and MCS patients exhibit similarly preserved arousal, but while VS/UWS patients remain largely unresponsive (with comatose patients also having their eyes closed), MCS patients show fluctuations in their behaviour, exhibiting temporal windows where volitional behaviour can be inferred (Bodien et al., 2022; Giacino, 2004, 2002). In the acute stage, prognosis is variable and highly dependant on the severity of the current state. Prognosis for chronic DOC patients is typically poor, with many patients remaining chronically unresponsive, and often dying without regaining consciousness (Estraneo et al., 2020). Treatment options remain limited, and rates of success are modest at best, although vigorous research is taking place to provide alternatives (Schnakers, 2017).

This challenging situation is perhaps not surprising, when one considers that even clinically distinguishing between VS/UWS and MCS patients is far from trivial: on the contrary, this diagnosis can be challenging for physicians without specialist training, and misdiagnosis rates reach up to 43% (Schnakers et al., 2009). Misdiagnosis can have important consequences, such as inadequate pain management, prognosis underestimation and even improper end-of-life decisions. Moreover, even when correctly performed, behaviour-based diagnosis is not fully accurate and might erroneously label some patients who in fact retain covert awareness. Functional brain imaging (fMRI) studies on VS/UWS patients provide evidence for covert intentional brain activity (Monti et al., 2010; Owen et al., 2006) in 10–15% of unresponsive patients and, in a few cases, similar methods have been used to enable simple functional communication with these patients (Claassen et al., 2019; Monti et al., 2010). Therefore, there is growing consensus for a need to go beyond behavioural observation alone, which has been matched by increasing availability of neuroimaging techniques which provide insights about patients’ brains: structural and diffusion MRI to identify the impact of lesions on anatomical structures and their connections, but also functional MRI, electroencephalography (EEG) (Naci et al., 2017) and functional near-infrared spectroscopy (fNIRS) (Abdalmalak et al., 2021) to observe brain activity, and positron emission tomography (PET) measures of metabolism (Bodart et al., 2017; Golkowski et al., 2017; Hermann et al., 2021; Sala et al., 2021) and receptor density (Qin et al., 2015) to name just a few.

The question therefore arises: can we capitalise on this wealth of data and decades of research to inform diagnosis in the clinic, and predict prognosis and devise more suitable treatments?

### 1.2 Why modelling?

Neuroscience is witnessing vigorous modelling efforts, from the scale of single neurons to whole-brain models (Amunts et al., 2022; D'Angelo and Jirsa, 2022; Einevoll et al., 2019; Roland et al., 2019). One way to understand what we mean by “model” is “a quantitative specification of a theory about how some aspect of the world works”. That can be as simple as a linear model describing the correlation between two variables; or it can take a more complicated form, such as coupled differential equations describing the evolution of a system. It is clear that obtaining quantitative models of DOC would be valuable not only in terms of advancing our scientific understanding of consciousness and its neural bases, but also in the clinic: for making more accurate diagnoses based on quantitative evidence, and for having a more robust understanding of the pathology's trajectory in order to try and steer it towards more favourable outcomes.

The first, crucial step towards modelling is therefore the identification and quantification of relevant properties. In the recent decades, with the advent of modern functional neuroimaging techniques, neuroscience has increasingly been able to identify links between loss of consciousness - whether pathological or pharmacological - and altered properties of brain activity and its dynamics (Afrasiabi et al., 2021; Barttfeld et al., 2015; Bonhomme et al., 2019; Campbell et al., 2020; Demertzi et al., 2019; Gutierrez-Barragan et al., 2021; Huang et al., 2020; Hutchison et al., 2014; Luppi et al., 2019; Panda et al., 2022; Redinbaugh et al., 2020; Song et al., 2018). Combined with increasingly detailed information about the healthy brain's macroscale structural and functional, microstructural, and molecular organization (Markello et al., 2022), this information can be leveraged by statistical models, such as machine learning (ML) algorithms, to characterise and categorise patient groups and sub-groups, but also to identify the features that best relate to DOC pathophysiology.

The ultimate end-goal of any DOC intervention is arguably to restore consciousness by re-establishing appropriate brain activity patterns (assuming a direct causal link from brain activity to consciousness). Descriptive statistical models that summarise the data along specific dimensions (whether in the form of Generalised Linear Models, or via machine learning methods), can reveal features of brain activity that are altered in DOCs. However, they are limited in their ability to predict how external manipulations might interact with neural circuits. For this purpose, it is necessary to develop generative models that allow *in silico* (i.e., through computational modelling rather than *in vivo*) exploration of hypothetical therapeutics aiming to rebalance brain activity in DOC patients. Generative models serve to describe how a system behaves under certain conditions or in response to perturbation. A major promise of generative models of brain activity is that they can be systematically and reversibly perturbed, making it possible to probe *in silico* interventions that are still beyond the capabilities of experimental research — whether in humans or animals (Cabral et al., 2017a; Cofré et al., 2020; Kringelbach and Deco, 2020; Shine et al., 2021). For these reasons, generative computational modelling of brain activity is gaining traction as a tool of choice for investigating the causal mechanisms that drive brain activity in both healthy and pathological conditions, complementing experimental research (Cabral et al., 2017a; Cofré et al., 2020; Kringelbach and Deco, 2020; Shine et al., 2021; Ramezanian-Panahi et al., 2022). However, current generative models of brain activity remain rather abstract, and their predictive power and fidelity to consciousness remain to be validated with experiments *in vivo*, before they can be translated into clinical applications (Kurtin et al., 2023). Once sufficient knowledge is reached regarding the key features of brain activity that reflect consciousness and how these can be modulated (first *in silico* then *in vivo*), novel avenues in addressing DOC can emerge.

**Fig. 1:**
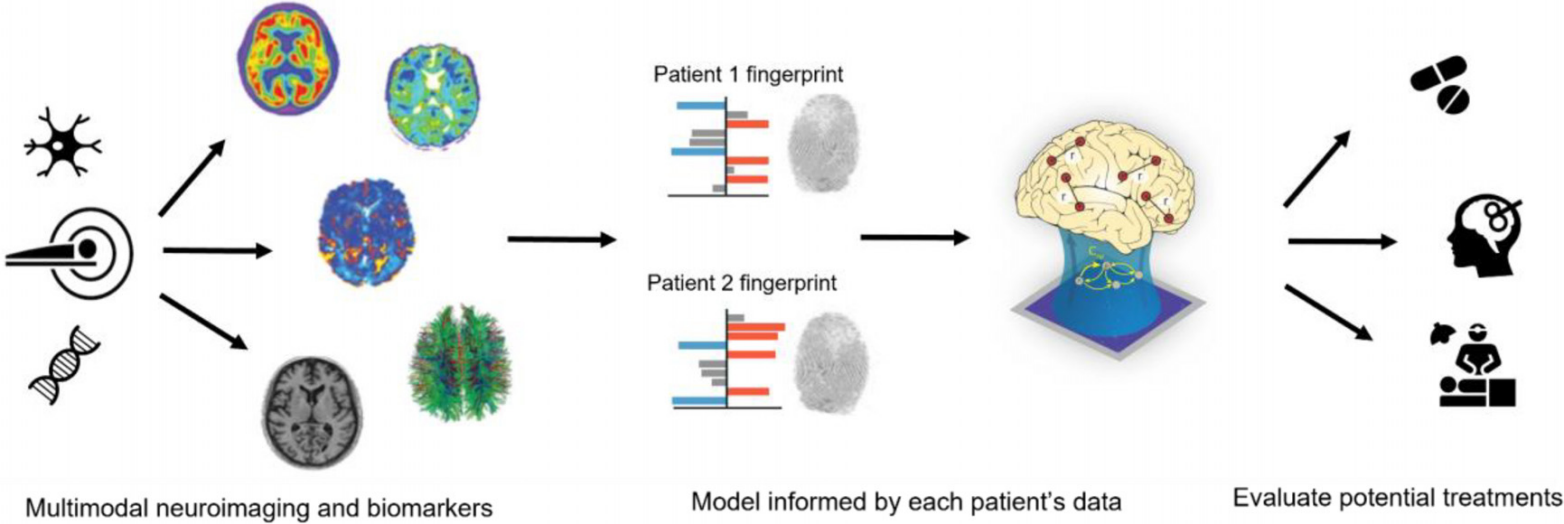
**Overview of “Phase 0 clinical trial” approach to modelling DOC.** Multimodal neuroimaging data from each individual patient are combined into a patient-specific “fingerprint”, and subsequently used (possibly together with normative data from the population) to inform a personalised brain model for each patient. For a given patient, the effects of different treatments can then be simulated *in silico* with their individualised model, to obtain insights about promising treatment avenues.

Ultimately, the aspirational goal for a mature field of DOC modelling is one of personalised medicine, where models of the healthy brain can be obtained from comprehensive subject-specific multimodal data at the individual level, informed by aggregate data about relevant features from the broader population, and then perturbed to match each patient's unique patterns of structural organization and neural signatures. Thereafter, the disorder-mimicking model can be used as an *in silico* test-bed (i.e., a “digital twin” of the patient (Erol et al., 2020)) to predict the outcome of alternative interventions and define the optimal therapeutic strategy for each patient aimed at restoring consciousness, operationalised as displacing the modelled activity in the general direction of restored consciousness and cognitive function (Fig. 1). The prospect of personalised computational models enabling this kind of “Phase 0 clinical trials” is especially appealing for DOC, because patients can vary widely in terms of aetiology, lesion site and extent, and symptoms - in turn calling for different treatment avenues with no one-size-fits-all approaches.

Here, we survey how different modelling approaches are being employed to address disorders of consciousness; we outline the gaps that exist between the current state-of-the-art, and the aspirational goal of a mature field of DOC modelling capable of finding application in the clinic; and we propose how some of these challenges could be addressed, to bring the field closer to this ambition.

## 2 Modelling approaches: lay of the land

To clarify how we hope for the field of DOC modelling to develop, it is of course necessary to outline not only what we wish to achieve, but also how close we currently are to achieving it. In turn, this requires an outline of current modelling approaches. In a rapidly developing field at the intersection of disciplines that are themselves rapidly evolving, such a taxonomy is a Sisyphean task, already outdated the moment it is written. However, our aim with presenting this taxonomy is not one of exhaustive enumeration; rather, below we provide a general map, omitting the details of the territory to more clearly convey a sense of the general landscape.

**Fig. 2:**
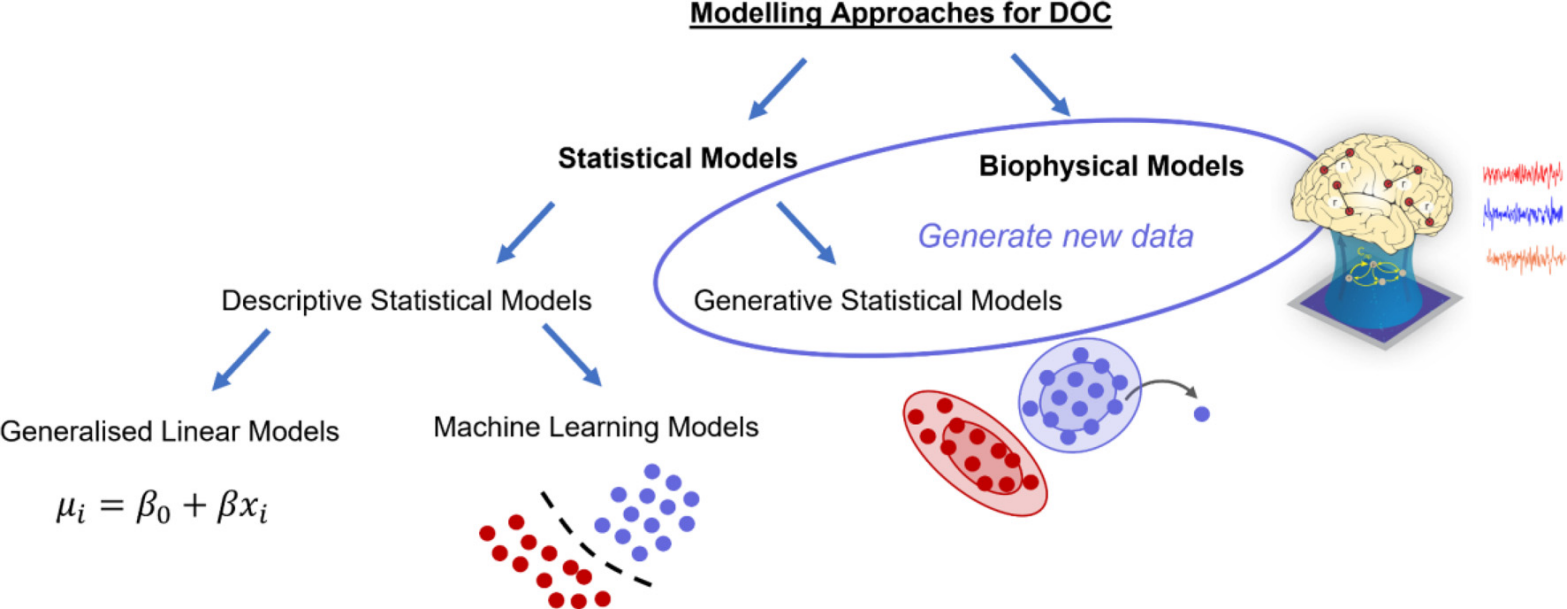
**Overview of relationships between distinct modelling approaches**. Biophysical computational models and Generative Statistical Models can be grouped together because they can be used to generate new data.

With this caveat out of the way, a first distinction to be made is between statistical models and biophysical computational models (Fig. 2). Statistical models can be subdivided based on whether they seek to characterise observed data in terms of summary statistics (descriptive models), or they simulate the data-generating processes, in order to create new instances (generative models). Descriptive models find widespread use for hypothesis testing in the form of Generalised Linear Models, to quantify correlations or group-level differences in terms of some dimension(s) of interest: “Is there a statistically significant difference between patients and controls in terms of measure X?”. However, descriptive statistical models can also be used in a hypothesis-free approach through machine learning techniques, to identify data-driven clusters or features that best discriminate between clinically relevant categories: “Based on this set of neuroimaging/clinical features, can we find previously undetected sub-groups of patients? Along what dimension are patients most discriminable from healthy controls?”. Generative statistical models explicitly learn how the data are distributed, enabling such models to generate new instances that are consistent with relevant statistical properties of the observed data. Of note, here we contrast generative with “descriptive” models, rather than with “discriminative” models; we use this broader term because we deal with a correspondingly broader class of statistical models than just classifiers intended to discriminate between categories.

In contrast, biophysical(ly-inspired) models in computational neuroscience employ equations derived from the underlying biophysics of neural activity (the physical, chemical and/or biological processes governing the dynamics of the system, at some suitable level of simplification and abstraction) to simulate the time evolution of the system, typically in the form of a system of differential equations (Breakspear, 2017; Shine et al., 2021; Ramezanian-Panahi et al., 2022). Note that such models also allow the generation of new data instances: not by learning the underlying probability distributions, but rather via direct simulation of the process that generated the data. By modelling the data-generating process rather than only the resulting data, biophysical computational models provide an avenue to test and evaluate possible causal interventions, addressing counterfactual questions about what would happen if some aspects of the process were disrupted or altered (e.g., “What happens if this connection is lesioned?”; “What happens if inhibition is increased?”). Generative models of brain activity have been shown to approximate the nonlinear response to different types of perturbations, such as electromagnetic stimulation, structural lesions, or psychoactive compounds (Burt et al., 2021; Deco et al., 2018a; Luppi et al., 2022c). With the caveat that experimental manipulation could be considered as the only true arbiter of causality, biophysical computational models are perhaps the closest in theory.

Below, we describe each of these model families in more detail.

### 2.1 Statistical models

#### 2.1.1 Descriptive statistical models

Descriptive statistical models comprise a large family, ranging from simple General Linear Models, to ML approaches (e.g. deep neural networks) that can identify discriminative features in the data. General Linear Models are perhaps the simplest form of descriptive statistical modelling, where a statistical relationship is hypothesised and then tested between two (or more) features of interest - for instance, some aspect of brain activity or anatomy versus diagnosis. This approach is widely used to identify predictors/correlates of disease severity or recovery, or to assess differences between patients and controls, or between sub-groups of patients. These models are mostly ways of testing specific hypotheses about the data (Demertzi et al., 2015; Luppi et al., 2021a; Lutkenhoff et al., 2020).

More recently, ML efforts are focusing on developing algorithms that learn statistical regularities in the input training data (defined by a set of features) in a more hypothesis-free manner, in order to make predictions on unseen data (e.g., diagnostic or prognostic predictions) (Bareham et al., 2018; Campbell et al., 2020; Chennu et al., 2017; Engemann et al., 2018; Hermann et al., 2021; Riganello et al., 2018; Stefan et al., 2018; Wielek et al., 2018; Zheng et al., 2017). Such algorithms can be as simple as a logistic regression and as complex as deep learning techniques. It would be beyond the scope of this article to describe all possible types of ML approaches. Briefly, the most relevant distinction for current applications is arguably between supervised (including semi- and self-supervised flavours) and unsupervised ML models. Supervised models are given ground-truth information, e.g., clinical labels about the diagnosis or outcome or treatment response of each subject, and their task is to find features in the neuroimaging data that best tell the data-points apart: that is, they perform classification or regression tasks. Unsupervised models instead typically seek to cluster data-points based on their features, to reveal similarities and differences by learning the statistical structure of the input data in the absence of predefined labels (Khosla et al., 2019).

Descriptive statistical models can also be characterised by the various input features considered. First, in terms of estimating the predictive power of single features or the optimal combination of multiple features (i.e., univariate versus multivariate analysis). Second, by the type of features used (e.g., unimodal features - single neuroimaging modality - or multimodal features - multiple modalities). Third, descriptive statistical models can vary in terms of the “feature engineering” strategy adopted: namely, investigators may pre-select features based on theoretical predictions (possibly after complex data-processing steps), or they may choose a more data-driven approach to feature selection. Since descriptive modelling approaches produce classification or regression, they can be used to determine the relevance of a given feature, or identify the most important feature(s) out of many, or classify data-points into controls or patients, or into patient sub-groups.

Note that another kind of “statistical model” can be identified: namely, “null” models that are used to test whether a given observation is statistically unexpected, not against the healthy population or a different patient sub-group (as is typically done when using descriptive statistical models to study DOC), but rather against some hypothesised process. We will not address this kind of model here (nor the related class of generative null models), but we refer the interested reader to an excellent recent review by Váša and Mišić (2022).

#### 2.1.2 Generative statistical models

Generative statistical models aim to reproduce the system under study or some of its (statistical) features, helping to understand what makes the recorded signal behave the way it does. These models are based on function approximation, utilising random processes that have been shown to – or are thought to – describe biological and brain data reasonably, to obtain “new” data that are consistent with statistical properties of the empirical data. Examples of these models are those based on Markov processes, which describe the evolution of a dynamical system by the probabilities of transitioning between separable "sub-states" of activity. From this perspective, each recording of brain activity can be interpreted as a series of transitions between such sub-states. Different approaches have been proposed to decompose brain activity detected with fMRI into a subset of states, including clustering algorithms, Hidden Markov Models (HMMs) or more advanced manifold learning algorithms (Busch et al., 2023). With HMMs, each sub-state is associated with a set of parameters of the observed data, with the most common choice being a multivariate autoregressive (MAR) model (Ou et al., 2015; Vidaurre et al., 2017, 2016). Alternatively, the activity or connectivity patterns observed over time can be clustered into a reduced set of clusters (or sub-states) and the dynamics can be similarly analysed as a Markov process by the transition probabilities between sub-states (Allen et al., 2014; Preti et al., 2017; Vohryzek et al., 2020). Overall, although the optimal approach to define brain sub-states remains under debate, the approach to characterise brain dynamics from fMRI data as trajectories in a state space (i.e. as a Markov process) has revealed high sensitivity to differentiate across a wide range of conditions, namely between sleep stages (Stevner et al., 2019), between controls and patients with psychiatric symptoms (Zarghami and Friston, 2020; Alonso Martínez et al., 2020; Farinha et al., 2022), between cognitive traits (Cabral et al., 2017b; Vidaurre et al., 2017; Uddin et al., 2021), and in psychedelic-induced altered states of consciousness (Lord et al., 2019; Olsen et al., 2022).

Note that these models are agnostic to the underlying (bio)physics; in other words, they do not seek to simulate the process that leads to the data, but only the statistical features of the data. Therefore, as with other kinds of generative statistical models, the mechanisms within these models may be biologically implausible, even though their outputs match the statistical features of measured data - an important distinction with biophysical computational models.

#### 2.1.3 Statistical models of DOC: current approaches

The diagnosis of consciousness in patients with DOC poses important challenges, and relying solely on clinical assessments of behaviour has limitations. Recent findings indicate that complementing clinical behavioural assessments with statistical neuroimaging analysis can improve the diagnosis accuracy and the evaluation of intervention outcomes. Various statistical approaches have been proposed to identify and classify patients with DOC using data obtained from different neuroimaging techniques, which could complement systematic behavioural assessment and help reduce the misdiagnosis rate reported in these patients. These methods encompass extracting markers from electrophysiological and neuroimaging data, and using them for multivariate statistical models, aiming to obtain insights on diagnostic and prognostic measures.

Sitt and colleagues used feature engineering to quantify high density EEG (hdEEG) putative neuronal signatures of consciousness (such as interareal connectivity, complexity, spectral activity, as predicted by current theories of consciousness) and quantify their performance to predict the state of consciousness of DOC patients (Sitt et al., 2014; King et al., 2013). They also used ML (support vector machine classifiers) to optimally combine those features and demonstrate that they carry independent predictive information. Specific patterns of resting brain connectivity measured through hdEEG have been found to strongly correlate with the re-emergence of consciousness after brain injury (Bareham et al., 2018). Machine learning analysis of sleep patterns using EEG has also been shown to accurately predict the level of consciousness in patients with DOC (Wielek et al., 2018). Graph theory has been applied to spectral connectivity estimated from EEG, and key quantitative metrics of these networks have been found to correlate with the continuum of behavioural recovery in patients with DOC (Chennu et al., 2017).

A deeper evaluation of EEG-based diagnosis of DOC patients performed by Engemann et al. (2018) who depicted an automated procedure that was suitable for cross-site and cross-protocol diagnosis of DOC. Based on ensembles of decision trees, they concluded that fluctuations in the power of theta and alpha EEG frequency bands were the most consistent and relevant markers. In line with these results, Stefan et al. (2018) showed that the power in alpha frequency band was the most effective at distinguishing patients in minimally conscious state (MCS) from those in unresponsive wakefulness syndrome (UWS/VS), while the average clustering coefficient obtained from beta-band coherence networks was the best predictor of outcome.

Resting-state fMRI (rs-fMRI) has also been used to identify differences in local, regional, and network activity between DOC patients and healthy controls. Machine learning models trained to distinguish between conscious wakefulness and anaesthetic-induced unconsciousness were investigated for their ability to identify pathologically induced unconsciousness (Campbell et al., 2020). The models achieved reliable performance within and across datasets and demonstrated potential for discriminating between degrees of pathological unconsciousness in clinical patients. Analysing rs-fMRI from the perspective of a trajectory in a state space, where the states were defined by clustering instantaneous patterns of phase coherence between brain areas detected over time, revealed that UWS/VS patients show primarily a brain pattern of low interareal phase coherence, with reduced transition probabilities (meaning that this state is more stable) when compared with healthy individuals and minimally conscious patients (Demertzi et al., 2019). In turn, the latter exhibit higher predominance of patterns in which brain regions activate in anti-phase, and switch more often between states.

At the structural level, Annen et al. (2018) used T1-weighted MRI images to extract regional brain volumes of white and grey matter, which were later used in a ML model to diagnose DOC patients. Machine learning based on diffusion MRI tractography was used to identify regions along the tracks that were most informative in distinguishing amongst DOC patients in distinct groups: UWS/VS, and two sub-groups of minimally conscious state, termed MCS+ and MCS- (Zheng et al., 2017). These results indicated that thalamo-cortical connections play a role in patients' behavioural profile and level of consciousness, and diffusion tensor imaging combined with ML algorithms could potentially facilitate diagnostic distinctions in DOC.

Moreover, multimodal approaches have been proposed to study cross-modal relations with respect to diagnosis and prognosis of DOC. In Hermann et al. (2021), the authors combined FDG-PET and EEG-based classification used based on a support vector machine to optimise diagnostic performance and predict 6-month command-following recovery in DOC patients (Hermann et al., 2021). A more recent work provided a systematic comparison of EEG-extracted features, visual interpretation as well as functional connectivity from rs-fMRI in models to diagnose DOC in the intensive care unit (Amiri et al., 2023).

Finally, statistical models have detected relevant markers for DOC in other physiological signals extending beyond the brain, such as in heart rate. Indeed, it has been shown that electrocardiography can also be used to diagnose DOC (Raimondo et al., 2017), providing partially independent information from the EEG signals. In a later publication, Riganello et al. (2018) showed that heart rate variability (HRV) entropy analysis, specifically the “complexity index”, can serve as a feature for differentiating between VS/UWS and MCS patients. Similarly, Candia Rivera and colleagues demonstrated that features extracted from the heart-evoked potential can be optimally combined to predict the state of consciousness of patients in resting state and during a task (Candia Rivera et al., 2021, 2023). These studies suggest that heart rate monitoring can provide an easy, inexpensive, and non-invasive diagnostic tool for disorders of consciousness, with the aid of statistical modelling techniques.

However, it is also important to clarify that these models have certain limitations. First, several of the previous works do not fully validate the models on new datasets and lack performance estimates beyond the initial cross-validated performances, which are often overestimated (Varoquaux, 2018). Second, their utility as part of clinical decision systems remains unknown. While the models provide novel insights into the DOC population, it is not yet clear what is the cost versus benefit of using such models. Further holistic analysis considering the human, technical and economic cost are required. Finally, and most importantly, statistical models of DOC are well suited to identify relevant features out of many possible candidates, but they do not explicitly model the process by which such features come to be relevant, i.e., they do not, on their own, provide insight on how to act on them, so they provide little insight about clinical interventions.

### 2.2 Biophysical and biologically-inspired computational models

#### 2.2.1 A vast space of biophysical models

Complementing purely statistical modelling techniques, *in silico* simulations of brain activity represent a powerful set of tools to study macroscale mechanistic questions in neuroscience (Cabral et al., 2017a; Cofré et al., 2020; Deco and Kringelbach, 2014; Kringelbach and Deco, 2020; Ramezanian-Panahi et al., 2022). Biophysical and biophysically-inspired computational models incorporate some aspect of biology (e.g., anatomical connectivity, excitatory and inhibitory populations etc..), and exist on a continuum, with varying biological plausibility and varying complexity and detail, which depends in part on the scale of the system being modelled (synapse, cell, region, whole brain network). As the terminology varies, we include in this admittedly broad category models that produce simulations of brain activity over time; such models also typically incorporate empirical data in the simulation process, such as the empirical connectivity between brain regions.

To date, there is no single model that can reproduce all the myriad aspects of human brain activity that span multiple spatial and temporal scales. The pursuit of such a universal model poses overwhelming conceptual and computational challenges. For this reason, researchers typically employ different computational models that are shaped according to their specific research question. Such models can vary widely in terms of their complexity, neurobiological realism of inputs and outputs, and even the target of the modelling: some summarise brain activity via a single global parameter (for example, fitting the model to the point where the mean firing rate becomes unstable, or a marker of criticality), whereas others seek to reproduce aspects of regional activity, or inter-regional connectivity (e.g., the pattern of functional correlations between regions). Likewise, models can vary in the spatial scale of interest - from small neuronal populations to the entire brain - and the temporal scale, from millisecond-resolution electrophysiology to the infraslow fluctuations of the BOLD signal (Cabral et al., 2017a; Cofré et al., 2020; Kringelbach and Deco, 2020). There is also a great deal of heterogeneity regarding the level of biophysical plausibility of computational models - abstract (sometimes called “phenomenological”; Ramezanian-Panahi et al., 2022; Pathak et al., 2022; Kurtin et al., 2023) models trade-off biological specificity for a clarity of insight, however at the potential cost of solutions that betray the true processes occurring in the brain.

In turn, both hypothesis-driven and data-driven approaches to modelling can be employed: some researchers seek to find model parameters that best fit the observed data, whereas others seek to assess the effects of perturbing the models in specific ways. An example of the former kind is Dynamic Causal Modelling: this approach employs biophysical equations to model fMRI or EEG activity, but its principal use is not for generating new data, but rather for selecting between competing accounts of a given phenomenon (i.e., “model selection”) (Casey et al., 2022; Friston et al., 2019, 2003; Preller et al., 2019; Stoliker et al., 2022). An example of the latter kind is network control theory (Gu et al., 2015), which models activity through an autoregressive process based on the simplifying assumption of macroscopically linear dynamics. As a result of this simplification, network control theory can characterise the propensity of brain networks to steer brain dynamics in a desired direction, or support the spreading of perturbations (e.g., transcranial magnetic stimulation pulses or deep brain stimulation), and identify stimulation regimes capable of producing a desired activity state (Betzel et al., 2016; Cornblath et al., 2018; Gu et al., 2015; Kim et al., 2018; Lynn and Bassett, 2019; Medaglia et al., 2017; Singleton et al., 2022; Tang et al., 2020, 2017; Zarkali et al., 2020) (but see also Pasqualetti et al. (2019), Suweis et al. (2019), Tu et al. (2018)) for a discussion of this approach and its limitations).

#### 2.2.2 Biophysical models in action

This state of affairs generates a vast space of phenomena to be explained*,* model types, and investigative approaches. However, we believe that each of these aspects should be considered in light of one overarching question: what unique insights does a particular combination provide us?

For instance, models with greater biophysical realism (e.g., dynamic mean field, Jansen-Rit) are especially well suited to investigate the effects of neuromodulatory influences at the macroscale, since they take into account the presence of distinct excitatory and inhibitory populations (Deco et al., 2014, 2013). These approaches have become increasingly prominent thanks to the availability of empirical measurements of the cortical distributions of neurotransmitter receptors and transporters from *in vivo* PET (Hansen et al., 2022) and post-mortem autoradiography (Goulas et al., 2021; Zilles and Palomero-Gallagher, 2017), as well as the regional expression of associated genes from transcriptomics (Arnatkevic̆iute et al., 2019; Hawrylycz et al., 2012; Markello et al., 2021). Incorporating such biological information has led not only to more realistic models (Deco et al., 2021; Demirtaş et al., 2019; Luppi et al., 2022a; Müller et al., 2020), but also to models capable of simulating pharmacological interventions with a variety of different drugs, covering the range from psychedelics to anaesthetics (Burt et al., 2021; Coronel-Oliveros et al., 2023, 2021; Deco et al., 2018a; Kringelbach et al., 2020; Luppi et al., 2022c). However, we note that a high degree of biological realism is not mandatory for a modelling approach to be able to capture the effects of pharmacological interventions, as recently demonstrated e.g. with extensions of network control theory that incorporate receptor expression to simulate the effects of psychedelics (Singleton et al., 2022), and previous work simulating the effects of anaesthesia using generalised Ising models from statistical mechanics (Kandeepan et al., 2020; Stramaglia et al., 2017).

On the other hand, Hopf/Stuart-Landau and Kuramoto models are suitable for modelling the oscillatory character of brain activity, and study aspects such as synchrony and metastability of neuronal oscillations (Cabral et al., 2017a; Deco et al., 2017; Deco and Kringelbach, 2016; Váša et al., 2015). Some approaches (e.g. Jansen-Rit) allow for easier translation across different simulations of functional brain activity (fMRI and EEG) (Coronel-Oliveros et al., 2021) and others can provide more latitude for perturbation. For example, regional oscillations in a Hopf model can be made subcritical (i.e., their amplitude naturally decay over time) or supercritical (i.e., the oscillations are sustained with constant amplitude) (Deco et al., 2019; Hahn et al., 2020; Ipiña et al., 2020; Jobst et al., 2017; López-González et al., 2021). Overall, although it may be tempting to categorise modelling efforts in terms of their choice of model (model family), we believe that the emphasis should be on what the model does and does not reproduce about brain function, and what opportunities and insights it offers.

#### 2.2.3 Biophysical computational models of DOC: current approaches

Over the last decade, whole-brain models of network dynamics have shown promising potential to investigate the causes of altered brain activity detected across DOC. Considering the dynamics of brain areas interacting in the neuroanatomical network (with different degrees of biophysical realism), the simulated activity is found to reveal features qualitatively similar to experimentally-recorded brain signals.

In addition to models explicitly aimed at recapitulating DOC (Abeyasinghe et al., 2020; Luppi et al., 2022c; Sanz Perl et al., 2021), biophysical models have also been employed to study other relevant and related conditions, such as the effects of brain lesions in conscious patients (e.g., stroke, brain injury; Rocha et al., 2022; Favaretto et al., 2022), or the effects of loss of consciousness in the healthy brain (e.g., sleep, anaesthesia) (Deco et al., 2017; Stramaglia et al., 2017; Kandeepan et al., 2020; Ipiña et al., 2020; Hahn et al., 2020). Indeed, recent efforts have also been undertaken to explain the changes in brain function observed in DOC patients - typically capitalising on the comparison with anaesthetic-induced unconsciousness to distinguish lesion-specific and consciousness-specific effects. This has leveraged recent empirical work that demonstrated important similarities between the brain dynamics of DOC patients and those of anaesthetised individuals (Barttfeld et al., 2015; Bonhomme et al., 2019; Campbell et al., 2020; Cao et al., 2019; Demertzi et al., 2019; Golkowski et al., 2021, 2017; Gutierrez-Barragan et al., 2021; Huang et al., 2020; Luppi et al., 2019; Panda et al., 2022; Song et al., 2018) with subsequent extensions identifying common underlying neuromodulatory mechanisms (Spindler et al., 2021).

Such models have to date capitalised on the similarities and differences between DOCs and anaesthesia, incorporating empirical evidence about patients’ disrupted patterns of structural connectivity between brain regions (Abeyasinghe et al., 2020; Luppi et al., 2022c, 2023; Sanz Perl et al., 2021). For instance, Luppi and colleagues (Luppi et al., 2022c) showed that the dynamics of a dynamic mean-field model can be altered in comparable ways by a pharmacological perturbation (increase of model regional inhibition in proportion to the empirical distribution of GABA-A receptors, to simulate the effects of the GABA-ergic agent propofol) or by perturbing the structural connectome to be analogous to the connectome of DOC patients. This work sought to explain how different changes to the brain's normal functioning (transient pharmacological intervention versus chronic structural lesion) can lead to similar patterns of brain dynamics. Conversely, Sanz Perl and colleagues (Sanz Perl et al., 2021) demonstrated that the states of anaesthesia and DOC can be distinguished in terms of how responsive they are to external perturbations, with DOCs being more resistant to change - consistent with their persistent nature versus the transient nature of anaesthesia.

The focus on responsiveness to perturbations is no coincidence: one of the best-performing empirical methods to estimate an individual's residual consciousness, the Perturbational Complexity Index, relies on evaluating the EEG response to brief perturbations induced by pulses of transcranial magnetic stimulation (Lee et al., 2022; Casali et al., 2013; Casarotto et al., 2016; Rosanova et al., 2018; Sarasso et al., 2021). Therefore, several efforts have been underway that seek to reproduce this phenomenon *in silico* (Bensaid et al., 2019), including incorporation into the popular modelling framework of The Virtual Brain (Goldman et al., 2021). For instance, a recent study (Luppi et al., 2023) used a dynamic mean-field model to demonstrate that the structural network alterations of DOC patients are sufficient to induce less hierarchical propagation of spontaneous events (“intrinsic ignition” (Deco et al., 2017; Deco and Kringelbach, 2017)), a phenomenon that is also observed empirically in patients’ brains, and that correlates with compromised measures of network controllability. Although these models offer some mechanistic insight to explain the features that differentiate between conditions, they still lack precise predictive value and more efforts are needed to fully realise the potential of computational models of brain stimulation, in particular for the design of personalised stimulation strategies with increased effectiveness (Kurtin et al., 2023; Vohryzek et al., 2022).

Other modelling efforts have also sought to capture aspects of the recovery process, by focusing on how brain activity spreads on the connectome and how this changes as a result of perturbations that alter the network's topology (Vasa et al., 2015; Cabral et al., 2012; Deco et al., 2018b). For instance, it has been shown that recovery from DOCs induced by severe injury may depend on re-routing of functional connections whose structural connections are impaired, without necessarily changing the SC itself (Kuceyeski et al., 2016).

### 2.3 Mixed modelling strategies

#### 2.3.1 Combining models to overcome their specific limitations

At this point, it is worth acknowledging that in describing the different model types, we have inevitably highlighted their differences - but similarities of course abound. All models are in some sense data-driven: whether because they look for patterns in the data, or because they use data to determine model fit and model parameters. Moreover, the choice of model type and features (if applicable) determines what the model can provide, such that none of these models is completely data-driven.

More broadly, a modelling workflow may involve multiple model types, by combining descriptive and generative statistical modelling (e.g., by performing statistical comparisons based on features identified by ML), or by combining statistical and biophysical modelling. For instance, unsupervised k-means clustering (a type of descriptive statistical modelling that clusters data based on specific features) may be used to identify distinct brain-states that can then be tested for differences in terms of relevant features (e.g., state occupancy) through statistical General Linear Models (Barttfeld et al., 2015; Lord et al., 2019) or used to identify target features to fit a biophysical computational model (Deco et al., 2019; Kringelbach et al., 2020). In addition to ML, biophysical computational models can also be used to extract additional features for discriminative models, such as identifying the global coupling parameter *G* that enables a biophysical model to best fit each subject's empirical data, and then comparing groups based on this (Coronel-Oliveros et al., 2023; Deco et al., 2017).

As we described above, the application of ML algorithms to neuroimaging data shows great promise for classifying physiological and pathological brain states. However, classifiers trained on high dimensional data are prone to overfitting, especially for a low number of training samples. To overcome this roadblock, over the last years strategies were developed that combine whole-brain computational models with statistical models (Vohryzek et al., 2022). The main rationale behind these strategies is to take advantage of the generative capabilities of the whole-brain model to meet the requirements that the statistical models have, in terms of the amount of data required to achieve sufficient statistical power and make generalisable predictions. In this vein, Arbabyazd and colleagues developed whole-brain models to create surrogate data to train random forest and Boost algorithm ML models to classify Alzheimer's disease patients and healthy participants (Arbabyazd et al., 2021). The authors demonstrated that the performance of both classifiers is comparable with that obtained when the models are trained with empirical data. Another strategy that combines both whole-brain and statistical models is postulated by Gilson and colleagues (Gilson et al., 2019). The whole-brain model fitting procedure generates model parameters, and the resulting effective connectivity between brain regions (quantification of the influence of one region's activity over the activity of another) can be used to train statistical discriminative models to classify different brain states. This shows that the modelling types can be combined into a “virtuous cycle”, progressively refining the features and identifying which ones could be intervened upon. Generative models can also be used for data augmentation (i.e., a mathematical process to generate synthetic data with the purpose of augmenting the training dataset, thus enhancing the model's learning capacity) to help improve the discriminative models, so the synergy between the two approaches can go both ways.

#### 2.3.2 Mixed models of DOC: current approaches

Combining machine learning and descriptive statistical modelling, Demertzi et al. (2019) and Luppi et al. (2019) both used k-means clustering to identify multiple dynamic states of brain connectivity from functional MRI of DOC patients and healthy controls. Demertzi and colleagues then extracted the prevalence of each state, and through descriptive statistical modelling demonstrated that UWS patients spend a greater proportion of time in a pattern characterised by high coupling with the underlying structural connectivity, with smaller chances to transition between patterns. This study also shows the value of combining not only multiple modelling strategies, but also multiple imaging modalities. Luppi and colleagues instead focused on network properties of the different dynamic functional connectivity states identified by k-means clustering, demonstrating through descriptive statistical modelling that a brain state of high network integration is especially affected by loss of consciousness, both in DOC patients and anaesthetised individuals.

In recent work, Sanz Perl et al. (2020) proposed implementing whole-brain models as a dynamical model informed data augmentation procedure to create meaningful surrogate data keeping the spatiotemporal structure of the original data. In that work, a random forest classifier was trained to discriminate between sleep stages and wakefulness with surrogate data generated with whole-brain models fitted to individual and group average empirical data for different stages of the wake-sleep human cycle. In both cases, the classifiers showed good performance when evaluated against empirical data, demonstrating that statistical models can be trained with whole-brain model individual and group average synthetic data.

Dynamical model data augmentation procedure was also used to train unsupervised statistical models. For instance, in Perl et al. (2020) the authors trained a variational autoencoder (VAE) with synthetic functional connectivity corresponding to wakefulness and deep sleep to obtain a low-dimensional representation of brain states. This strategy allowed the authors to find an orderly trajectory from wakefulness to brain injured patients in a latent space whose coordinates represent metrics related to functional modularity and structure-function coupling, both increasing alongside loss of consciousness. These results suggest that other brain states (e.g., DOC) could be captured and understood in terms of trajectories within a low-dimensional latent space, with potential applications in diagnosis and prognosis.

## 3 Interim summary

**Fig. 3:**
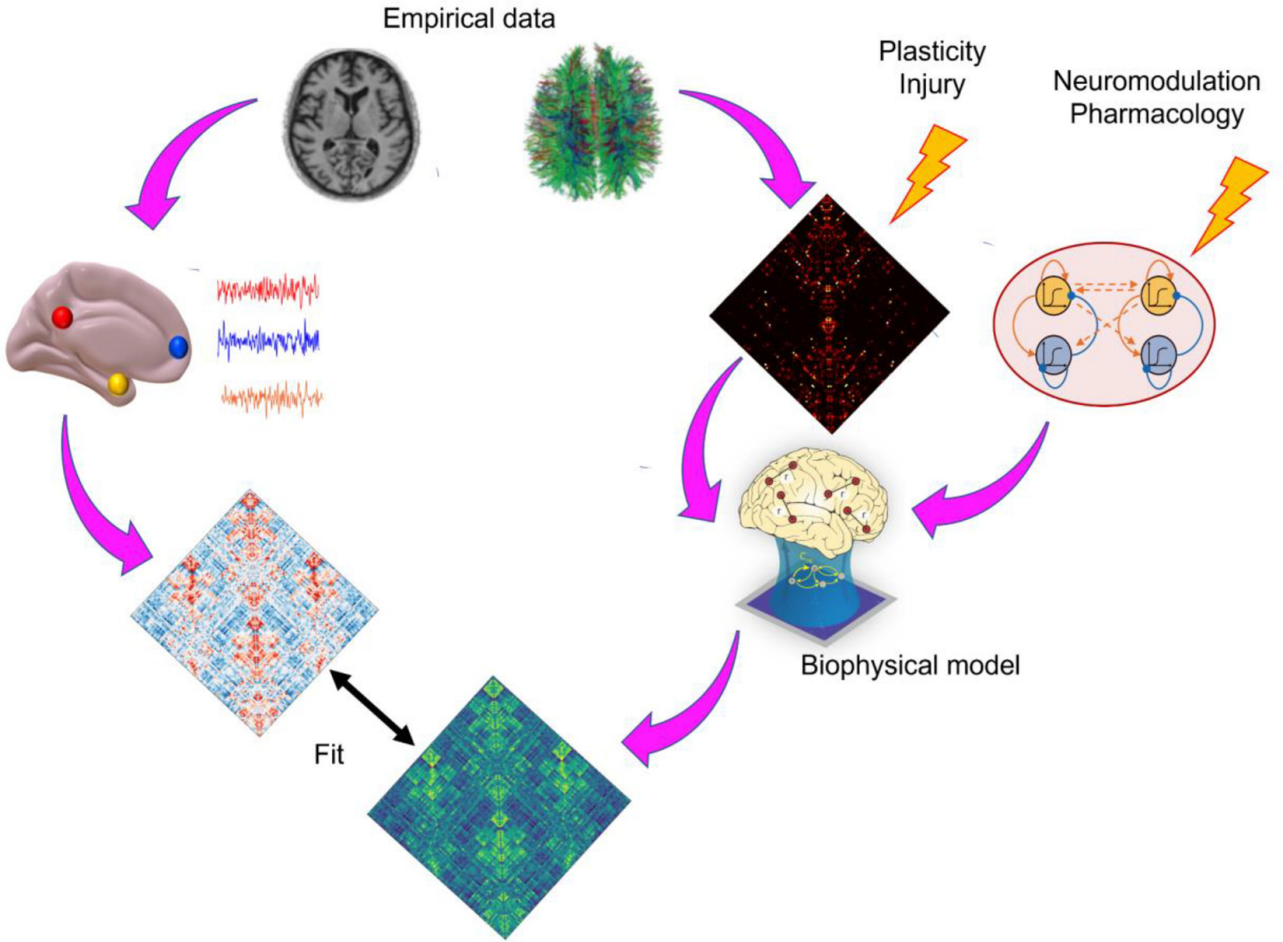
**Overview of using biophysical computational models to evaluate causal interventions**. Empirical structural connectivity between regions can be reconstructed from diffusion MRI tractography, which provides the global connectivity between regions. Perturbations can be applied to this connectivity, to simulate lesions or plasticity. Additionally, perturbations to the local dynamics of each region can simulate neuromodulatory influences and pharmacology. The resulting simulated activity and functional connectivity can then be compared against empirical data, to evaluate the model's goodness-of-fit.

Overall, while both generative statistical and biophysical computational models aim to simulate the outcome, only the biologically-inspired models aim to simulate the entire data-generating process. This causal flavour is derived from their biological inspiration - with the understanding that insight into causality should be rooted on models that are biologically realistic. This means that biophysical computational models are also well suited to providing insight about counterfactuals, by directly simulating the effects of an intervention, (Fig. 3) and therefore about the likely effects of a given treatment option. This stands in contrast with statistical models, which are most suited to diagnosis and prognosis. Of course, since generative statistical models learn the joint probability distribution of multiple features, it is possible to use such models to explore how variation in one feature would affect another. For instance, to test the effects of a certain drug, one could first assess how this drug modifies the features of interest in the data, and then explore the region of modelled data-space that corresponds to these changes. However, such out-of-sample generalisations are notoriously difficult. In contrast, having a causal model of how one variable influences the whole system's behaviour is precisely the *raison d'etre* of biophysical computational models, as such relationships can be explicitly encoded.

In other words, descriptive models are well suited to identify relevant features out of many possible candidates, but because they do not explicitly model the process by which such features come to be relevant, they do not on their own provide insight on how to act on them: they do not tell the clinician how to intervene. Generative statistical models and biophysical models can help to test possible interventions for the promising features identified by discriminative models: the foundations towards “digital twins”. The downside is that finding the appropriate level of biological complexity is not straightforward: a mismatch with reality will always be present, to some extent, and it can be challenging to determine whether it is innocuous abstraction, inevitable noise, or misleading mis-specification. However, as reviewed in the section on Mixed modelling strategies, research efforts have been ongoing to combine the strengths of both modelling approaches, and to use one to mitigate the shortcomings of the other, representing the source of numerous recent insights.

## 4 The road towards a mature field of DOC modelling

Having provided an overview of the different kinds of modelling approaches that are being employed to investigate disorders of consciousness, in this second part of our article we provide recommendations for how the field can move forward: the challenges that lie ahead, and how we believe that they can be overcome.

### 4.1 Open challenges in simulating DOC

Despite these favourable characteristics, applications of whole-brain modelling to DOCs are relatively recent, arguably because this endeavour involves substantial challenges. In effect, the complexity and heterogeneity of DOCs in terms of aetiology — with each patient typically presenting unique patterns of cerebral lesions and deficits — greatly complicates extrapolating modelling paradigms based on healthy brains.

One way to approach the challenge of modelling brain activity of patients with DOC is to decompose the problem into two more tractable questions: (i) modelling unconsciousness, and (ii) modelling brain damage. As testbeds to model unconsciousness, at least three candidates present themselves: the endogenous, transient state of unconsciousness comprising dreamless (deep) sleep; the endogenous but pathological unconsciousness of epileptic seizures; and exogenously induced unconsciousness resulting from the administration of general anaesthetics - which unlike sleep, is a perturbation of the brain's spontaneous state, in this sense resembling DOC more than sleep does. These conditions can also be studied in animal models, where there is enormously greater capacity for experimental access and manipulation, leading to substantial advances in our understanding of their neurobiology. In turn, this neurobiological knowledge makes it possible to formulate specific mechanistic hypotheses based on experimental results, and develop suitable computational models spanning from very biologically detailed ones (Ching et al., 2010, 2012) to more abstract ones that nevertheless capture well-known features of the anaesthetised or sleeping brain (Deco et al., 2017; Kandeepan et al., 2020; Stramaglia et al., 2017).

On the other hand, anaesthesia and sleep are typically studied in the healthy brain in humans, whereas DOCs typically involve lesions of varied extent and location, such that both grey and white matter can be affected. However, not all brain lesions result in chronic disorders of consciousness, and neuroscientists have developed computational models to understand the effects of brain damage on patients’ cognition and brain function. For example, previous studies have shown that computational modelling can provide specific predictions of cognitive impairment linking structural lesions and their effect on neurodynamics (Cabral et al., 2017a; Vasa et al., 2015; Rocha et al., 2022; Favaretto et al., 2022) — in particular highlighting the role of the integrity of “hub” nodes for the assessment of cognitive loss. For both models of unconsciousness and models of brain injury, animal models have played a fundamental role in shaping our understanding, thanks to their greater experimental accessibility (Beppi et al., 2023; Redinbaugh et al., 2020; Bastos et al., 2021; Tasserie et al., 2022; Barttfeld et al., 2015; Gutierrez-Barragan et al., 2021). Studies in anaesthetised macaques provided the first evidence that brain structural and functional connectivity become more similar when consciousness is lost (Barttfeld et al., 2015) - which was later confirmed both in mice (Gutierrez-Barragan et al., 2021) and humans (Demertzi et al., 2019). More recently, several studies demonstrated that electrical stimulation of the central thalamus can restore neural and behavioural signatures of consciousness in anaesthetised macaques (Redinbaugh et al., 2020; Bastos et al., 2021; Tasserie et al., 2022), a feat which is possible thanks to animals’ greater experimental accessibility. Developing computational models of animal models (as done e.g. by Hahn et al. 2020) is therefore going to be a key stepping stone towards the human counterpart.

We note that in order to characterise the current gaps that need to be bridged, it is not sufficient to identify the current state of the art: one must also clearly define the goals to be achieved. Upon doing so, it becomes apparent that for the present endeavour, two different kinds of goals exist, which are closely aligned but nevertheless not identical. On one hand, there is the clinicians’ goal to improve diagnosis and prognosis and ultimately treat (or at least alleviate the suffering of) patients with DOC. On the other hand, there is the scientific goal of understanding how the brain can become stuck in a state of chronic unconsciousness, and how this illuminates the mechanisms that in the healthy brain enable consciousness to emerge from matter. The history of medicine is replete with examples of treatments that were discovered serendipitously or through trial and error, and hence employed before being fully understood. Arguably, the mechanisms of anaesthesia, one of the most important tools in medical history, remain incompletely understood - which fortunately does not prevent anaesthetists from using it to spare patients the intolerable suffering of surgery. Nevertheless, a greater scientific understanding can only benefit the clinical approach, both in terms of diagnosis and treatment. However, a scientist may be satisfied with more abstract understanding at the group level, whereas clinicians inevitably do not treat groups, but individuals - and they have a stronger incentive to provide timely answers, since they cannot indefinitely put off decisions about treatment avenues. Therefore, in this aspect the scientific and clinical goals diverge, and this divergence needs to be considered when outlining our desiderata as a community and how we intend to attain them.

In particular, the clinical and scientific perspectives may hold different views on the main trade-off involved in biophysical/generative modelling of DOC: biological realism versus complexity. If we want to have models that help us to predict outcomes or evaluate *in silico* the potential for different treatments, then a mis-specified model that fails to take into account relevant aspects of neurobiology (the definition of which is itself part of the problem, of course) may constitute a costly mistake. Likewise for the case where statistical/discriminative models are being used for diagnostic/prognostic purposes, or to identify patients for more in-depth investigation. This issue is intertwined with the modeller's perennial question: what counts as a well-fitting model? There are two parts to this question. On one hand, the model must be able to match the desired aspect of the data. But perhaps even more importantly, such an aspect (the loss function in terms of which the model is evaluated) must be properly identified. A model that faithfully reproduces the wrong function is at best useless, and at worst actively misleading. Finding a suitable objective function - in this case, a neuroimaging marker that a given brain is capable of entertaining consciousness - is a task for empirical investigations of consciousness, to be aided by discriminative statistical models.

With this in mind, we lay out four main aspirations for the modelling of DOC; for each desideratum, we also highlight what we see as the most pressing challenges, and we outline some potential approaches that we believe could contribute to address such challenges.

### 4.2 Desiderata for the future of DOC modelling

#### 4.2.1 Desideratum 1: greater generalisability

Models need to be able to generalise across individuals and across different aetiologies (e.g., traumatic versus anoxic/hypoxic injury). More broadly, models should generalise across different cohorts. Within the same individuals, models ought to be able to provide a match for a particular individual's multimodal data, reflecting their capacity to truly provide insight about the patients’ brains. Generalising to the broader population is the implicit goal of the statistical tests we employ, and ability to do so is a marker that we possess true scientific insight into a given phenomenon.

Building models that capture multimodal data can help to address the first challenge that we face when trying to improve generalisability: the need to identify relevant spatial and temporal scales that our models should aim to capture. For instance, fMRI and EEG differ in terms of both spatial and temporal resolution, so a model that can simulate both would imply the ability to capture both slow and fast dynamics. Additionally, models that can provide a good fit to multiple modalities (e.g., both fMRI and EEG data) provide intrinsic validation for their biological plausibility. This avenue of addressing the first challenge to generalisation can capitalise on the existence of rich datasets about patients, spanning not only neuroimaging but also neurotransmitter expression, transcriptomics and proteomics, which are now becoming increasingly available. Of course, the success of this endeavour depends on the data being compatible and of high enough quality, to make sure that the additional data are not merely adding noise. In particular, multi-modal models may best enhance generalizability when cross-modal data at different scales are effectively integrated.

A second challenge to generalisation is that DOC patients vary widely not only in terms of diagnosis, but also in terms of lesion extent and location, as well as severity and aetiology. This means that the boundaries for generalisation remain ill-defined: which subset of patients constitute an intended target for generalisation (such that failing to generalise to such patients indicates a shortcoming of the model), and which patients are instead simply beyond the scope of the model? To address this challenge, a useful starting point may be to identify homogeneous sub-groups of DOC patients with similar diagnosis and similar aetiology, and use this sub-group-level as a stepping stone between the individual-level and the broader group-level. In particular, it may be especially fruitful to focus on patients displaying similar constellations of symptoms, and building models that can be differentially perturbed to obtain individual-specific symptomatology.

An intriguing potential avenue for generalisation is the recent proposal that late stages of dementia may be behaviourally equivalent to DOC (Huntley et al., 2021). If so, this would provide an invaluable additional source of information, since the progression is gradual and observable, unlike most of the injuries that lead to current DOC patients’ admission. However, this avenue is itself not without challenges. Gradual atrophy of a region due to neurodegeneration need not produce the same cognitive and behavioural deficits caused by sudden ischaemic stroke or traumatic lesion of the same region. Therefore, the rate of change, and resulting role of plasticity, may point to distinct processes. Nevertheless, the greater similarity between gradual progression stages of brain degeneration in dementia may provide a suitable testing ground for models to generalise.

#### 4.2.2 Desideratum 2: patient-specificity

**Fig. 4:**
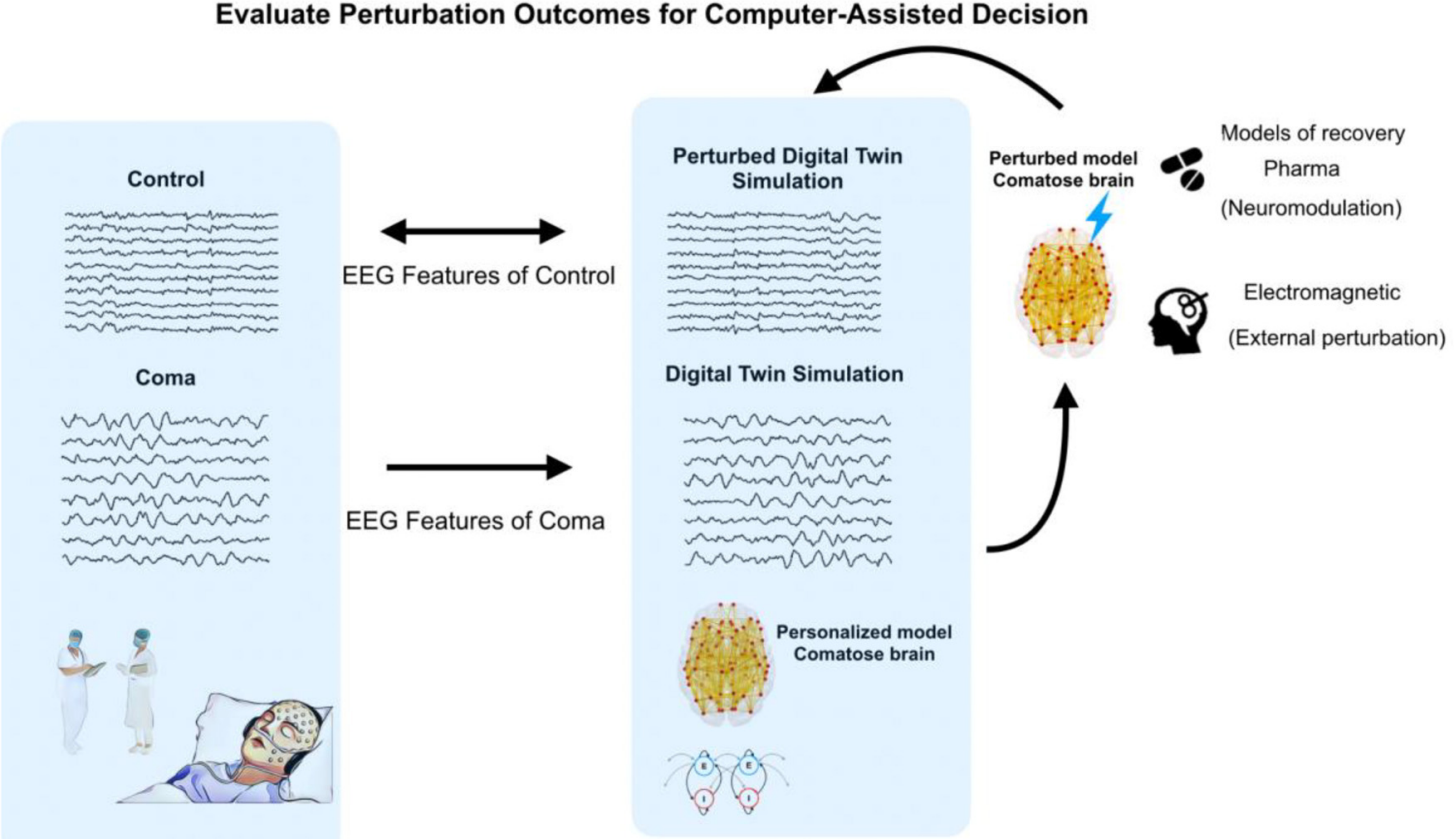
**“Digital Twin” approach.** The EEG data are used as an example application. The patient's data are fitted with a personalised brain model, creating a "digital twin" of the patient whose simulated activity best resembles the patient's empirical EEG. The effects of various interventions are then simulated on the “digital twin” and evaluated based on goodness of fit compared to normative data from healthy controls. The underlying assumption is that an intervention that makes the EEG activity of the “digital twin” resemble the EEG of control subjects may be a good candidate for a clinical intervention in the patient.

As anticipated above, the clinical need is to treat individual patients, and one of the greatest promises of modelling is the possibility to develop personalised models for each patient, in the vein of the “Digital Twins” paradigm (Fig. 4). This can broadly take two forms. Under the first approach, a “digital twin” of patient X is a model obtained from aggregating large amounts of data from other patients who resemble patient X in some relevant aspect, with the aim of triangulating on this specific patient. Under the second approach, a “digital twin” is built from the patient's own multimodal data. Of course, the two approaches are not mutually exclusive: group data can be used to “fill in” missing information about a patient in question.

This is especially valuable given the wide variability of DOC patients and their trajectories. Unfortunately, the risk is that models may end up fitting idiosyncrasies rather than capturing what is still common across DOC patients: their impaired consciousness. In other words, single-patient modelling risks overfitting to features unrelated to DOC (especially since each patient often presents a unique and complex aetiology, with a potential host of deficits and impairments that are not directly related to their unconscious status). Under such conditions, clinicians with an interest in simulating treatment options find themselves between the Scylla of using a group-level model that may not apply to their specific patient and his/her needs; and the Charybdis of using a personalised model, but with little confidence that it reflects generalizable information. Therefore, the main challenge to patient-specific models is to establish trust in single-patient predictions, mitigating the impact of overfitting and measurement noise.

Personalised models could be created, for example, using a transfer learning-like approach wherein the model begins by representing a population and is fine-tuned using an individual's observed data (Gu et al., 2022). These personalised models would then have to be tested in how well they reflect empirical data. This could be accomplished by successfully predicting a patient's trajectory, matching longitudinal data. A second, more demanding bar to clear would be to show that a model can successfully predict the effects of a given intervention on a patient. This could be done for patients who are already due to receive a given treatment (e.g., a specific medication). If replicable across patients, this would provide the kind of evidence that the model may also be suitable to inform the choice of treatment.

#### 4.2.3 Desideratum 3: models of recovery

The current state of DOC modelling focuses primarily on distinguishing between groups, with the goal being either diagnosis or obtaining a mechanistic understanding of loss of consciousness (or a combination of both). However, the prime clinical goal is to achieve recovery, and therefore, there is a need for models that can reflect the recovery process: both its spontaneous course (which would also aid prognosis), and how it can be promoted or accelerated through interventions. The main challenge to this goal is that few patients fully recover, and many instead decline; and for those who do recover, this process is typically very protracted and gradual, rather than an abrupt change (Edlow et al., 2020). If we do not understand how the brain recovers, we remain limited in our ability to help it to recover.

Conceptually, it cannot be expected that the process of recovery from DOC will mirror the process of loss of consciousness, as one might expect to happen for sleep or anaesthesia, because it is not possible to simply “un-do” the damage that a patient has suffered, in the way that anaesthesia wanes as the drug concentration is diminished. And in fact, even for sleep and anaesthesia there is growing evidence that loss and recovery of consciousness are actually asymmetric, with an “inertia” or hysteresis effect being observed for both, suggesting more complex transitions (Friedman et al., 2010; Kuizenga et al., 2018; Luppi et al., 2021b; Proekt and Hudson, 2018). Additionally, even if the process were symmetric, we do not have imaging data about the moment of the injury, in the same way that we can image anaesthetic-induced loss of responsiveness.

Nevertheless, some ways to mitigate these limitations can be envisioned. First, emergence from sleep and anaesthesia, though imperfect models, can still provide valuable insights about the general process of recovering consciousness. Second, in some cases it may be possible to scan patients before and after treatment: especially if such treatments were to show a degree of success (such as the effects that zolpidem on a small subset of DOC patients (Noormandi et al., 2017)), then the pre- vs post-treatment comparison could provide insights about emergence to inform modelling efforts. Third, longitudinal imaging can provide data about both gradual recovery and gradual decline, which can then be used as benchmarks for models intended to capture disease progression rather than just static snapshots.

Imaging acute patients can also provide clearer data closer to the time of loss of consciousness, while also providing the occasion to make model-based predictions that can then be evaluated against the actual progression of the patient (although the acute phase during intensive care is inevitably complicated by practical difficulties and confounds such as sedation). In this context, studies of recovery from stroke or TBI without loss of consciousness could provide an avenue to decouple brain injury and repair from their effects on consciousness, for instance in terms of spatial distribution of damage (Maas et al., 2022, 2014; Olafson et al., 2022, 2021), which is undoubtedly one of the key challenges. This approach can then be complemented by studies of loss and recovery of consciousness in animal models, where causal intervention is more feasible and there can be greater experimental access; recent work has been identifying promising stimulation targets to awaken non-human primates from anaesthesia, and modelling this scenario offers a clear avenue to make progress on the field's ability to model recovery of consciousness (Bastos et al., 2021; Donoghue et al., 2019; Tasserie et al., 2022). Together, these approaches may offer a way to devise models of recovery from DOC, by modelling different aspects of the recovery process, across multiple timescales. To this end, incorporating aspects of temporal evolution over the long term (e.g., plasticity of connections (Hellyer et al., 2016)) will be a key direction for the field.

#### 4.2.4 Desideratum 4: improved confidence

This last desideratum is not unique to modelling DOCs, but rather it is a broader one that is shared with modern applications of modelling and machine learning, especially in science and healthcare. Namely, models need *explainability, accountability, reliability,* in order to be used with confidence. Can the model provide an understandable explanation of the decision? Does it explain how and what it learnt from the data? Some work has already been carried out in this direction, by adopting interpretable deep learning approaches to the classification of sleep, anaesthesia, and pathological unconsciousness (DOC) based on EEG features (Lee et al., 2022). These authors showed that the model can disambiguate between awareness and arousal, correlating with intervention-based approaches.

This desideratum can be summarised by saying that “models must not be black-boxes” (Castelvecchi, 2016; Heinrichs and Eickhoff, 2020). To address these goals, some considerations need to be first taken into account. Firstly, model fitting: many of the smaller-scale biophysical computational models are ill-parameterized and may result in overfitting; and the same may also apply to statistical approaches:an issue that is also related to our desideratum about generalisability. Multiple avenues exist to address this challenge. On one hand, repeated studies on the same patients could help to boost confidence and increase the signal-to-noise ratio within individual patients. This could be combined with increased data-sharing to pool samples across different cohorts, which would not only contribute to generalisability, but also provide better ways to avoid overfitting via cross-validation. This approach would also increase the representation of each sub-group of patients - as well as potentially helping to identify relevant sub-groups for stratification. With data-sharing comes the need to harmonise data acquisition paradigms from different sites (Orlhac et al., 2022; Pomponio et al., 2020) as well as using homogeneous preprocessing pipelines and consistent fitting procedures across studies, to make research more comparable and facilitate identifying improvements over the state-of-the-art - which is typically not possible in current research, when studies differ in terms of model used but also cohort, data processing, and fitting criterion.

## 5 General recommendations

In addition to the Desiderata outlined above, there are also a number of more general recommendations that we believe would help both the study of DOCs as a whole, and the more specific endeavour of modelling DOCs.

First, adding to the call for data-sharing and harmonisation, we believe that DOC research should take inspiration from the non-human primate neuroscience. Like most DOC datasets, non-human primate neuroimaging datasets tend to comprise a relatively small number of individuals, due to the difficulties of data collection. In non-human primate neuroscience, one way to overcome this limitation has been by repeated data acquisition from the same individual, to boost the signal-to-noise ratio. To the extent that this is feasible for DOC patients, we believe that availability of multiple scanning sessions, both with the same and with different neuroimaging modalities, would be of great help to the modelling effort. Additionally, the field of non-human primate neuroscience has recently come together to organise large-scale data-sharing initiatives to accelerate research, and we hope for a similar initiative for DOC (Michael Milham et al., 2018).

A second avenue for progress in DOC modelling research will be to combine not only different flavours of models, but also combine and iterate between theory- and data-driven approaches (Luppi et al., 2021). We envision that the development of *in silico* models that reproduce the ML-derived insights will be an essential component of a mature field of DOC modelling.

Third, it will be important to obtain a more comprehensive (and ideally, more formal) understanding of how the space of possible models matches onto the space of modelling goals, in the context of DOC. In other words: when does each model stop being applicable? As modelling and model types evolve, so will this landscape change.

Finally, with new technologies inevitably come new considerations pertaining to their ethical use. In particular, we have advocated for the prospect of using models to assess the expected outcome of a given treatment option, for a given patient, in the paradigm of “digital twins” (Vohryzek et al., 2022). The question arises, however: If modelling suggests that a treatment is likely to be ineffective, to what extent should this be sufficient grounds for not attempting the treatment? We expect that there is no one-size-fits-all answer to this question: rather, as models become more personalised and their predictive power increases, they may become a greater component of the broader cost-benefit evaluation pertaining to each patient. We do not expect that such models will replace clinicians’ assessment, nor do we think that this would be desirable: rather, they will provide clinicians with additional information when forming their expert judgments.

## 6 Concluding remarks

Overall, modelling approaches are perhaps one of the most promising avenues of progress in our understanding of disorders of consciousness, owing to the combination of increasing computational resources and increasingly detailed and powerful models. Although a number of gaps exist in the current state-of-the-art, we have outlined how the field could overcome these gaps to realise the full scientific and clinical power of computational modelling. The possibility of building “digital twins” and using them for “Phase 0 clinical trials” may substantially advance our ability to identify suitable treatments for individual patients, capitalising on the increasing amounts of multimodal neuroimaging data available.

In this context, we emphasise that there is a distinction to be drawn between modelling the difference between a conscious and an unconscious brain, and developing a full theory of consciousness (in terms of what consciousness “is”). We acknowledge that understanding consciousness is a challenging question to address, and a full understanding of human consciousness will require a concerted, multi-disciplinary approach well beyond the one outlined here: our goal is focused towards clinical relevance. Nevertheless, throughout the history of medicine, treatment has often preceded the detailed understanding of the ailment. We believe that a mature state of computational modelling of DOC will provide invaluable tools for personalised diagnosis, prognosis, and treatment, but we also believe that in the process of achieving this status, computational models will continue to provide important insights about consciousness itself.

We reiterate that our modelling taxonomy is not meant to be exhaustive or definitive. Indeed, our group of expert scientists and clinicians disagreed at times about the most suitable way to characterise the different model types, and which ones should be grouped together, and under what title. Are biophysical and statistical models “generative” in the same sense? Would it be more helpful to describe the main distinction between models as “top-down” (testing a given feature of interest) versus “bottom-up” (putting together the pieces to reproduce a behaviour of interest)? In some sense, the word “model” itself may simply hold different meanings for different researchers. We hope that this work will contribute to establishing a common ground and a common reference for discussing modelling approaches in the context of disorders of consciousness, so that clinicians and computationalists will achieve greater integration of different approaches, to overcome the challenges that we have outlined here, and achieve greater progress towards our shared goal: curing coma.

## Ethics statement

This work did not involve collection or analysis of data.

## CRediT authorship contribution statement

**Andrea I. Luppi:** Conceptualization, Writing – original draft, Writing – review & editing, Visualization. **Joana Cabral:** Writing – original draft, Writing – review & editing, Visualization. **Rodrigo Cofre:** Writing – original draft, Writing – review & editing, Visualization. **Pedro A.M. Mediano:** Writing – review & editing. **Fernando E. Rosas:** Writing – review & editing. **Abid Y. Qureshi:** Writing – review & editing. **Amy Kuceyeski:** Writing – review & editing. **Enzo Tagliazucchi:** Writing – review & editing. **Federico Raimondo:** Writing – review & editing. **Gustavo Deco:** Writing – review & editing. **James M. Shine:** Writing – review & editing. **Morten L. Kringelbach:** Writing – review & editing. **Patricio Orio:** Writing – review & editing. **ShiNung Ching:** Writing – review & editing. **Yonatan Sanz Perl:** Writing – review & editing. **Michael N. Diringer:** Conceptualization, Writing – review & editing, Supervision. **Robert D. Stevens:** Conceptualization, Writing – review & editing, Supervision. **Jacobo Diego Sitt:** Conceptualization, Writing – original draft, Writing – review & editing, Supervision.

## Declaration of Competing Interest

The authors have no conflicts of interest to declare.

## Data Availability

No data was used for the research described in the article.

## References

[bib0001] Abdalmalak A., Milej D., Norton L., Debicki D.B., Owen A.M., Lawrence K.S. (2021). The potential role of fNIRS in evaluating levels of consciousness. Front. Hum. Neurosci..

[bib0002] Abeyasinghe P.M., Aiello M., Nichols E.S., Cavaliere C., Fiorenza S., Masotta O., Borrelli P., Owen A.M., Estraneo A., Soddu A. (2020). Consciousness and the dimensionality of DOC patients via the generalized ising model. J. Clin. Med..

[bib0003] Afrasiabi M., Redinbaugh M.J., Phillips J.M., Kambi N.A., Mohanta S., Raz A., Haun A.M., Saalmann Y.B. (2021). Consciousness depends on integration between parietal cortex, striatum, and thalamus. Cell Syst..

[bib0004] Allen E.A., Damaraju E., Plis S.M., Erhardt E.B., Eichele T., Calhoun V.D. (2014). Tracking whole-brain connectivity dynamics in the resting state. Cereb. Cortex.

[bib175] Alonso Martínez S., Deco G., Ter Horst G.J., Cabral J. (2020). The dynamics of functional brain networks associated with depressive symptoms in a nonclinical sample. Front. Neural Circuits.

[bib0005] Amiri M., Fisher P.M., Raimondo F., Sidaros A., Cacic Hribljan M., Othman M.H., Kondziella D. (2023). Multimodal prediction of residual consciousness in the intensive care unit: the CONNECT-ME study. Brain.

[bib0006] Amunts K., DeFelipe J., Pennartz C., Destexhe A., Migliore M., Ryvlin P., Furber S., Knoll A., Bitsch L., Bjaalie J.G., Ioannidis Y., Lippert T., Sanchez-Vives M.V., Goebel R., Jirsa V. (2022). Linking brain structure, activity, and cognitive function through computation. eNeuro.

[bib0007] Annen J., Frasso G., Crone J.S., Heine L., Di Perri C., Martial C., Coma Science Group Collaborators (2018). Regional brain volumetry and brain function in severely brain-injured patients. Ann. Neurol..

[bib0008] Arbabyazd L., Shen K., Wang Z., Hofmann-Apitius M., Ritter P., McIntosh A.R., Battaglia D., Jirsa V. (2021). Virtual connectomic datasets in Alzheimer's disease and aging using whole-brain network dynamics modelling. eNeuro.

[bib0009] Arnatkevic̆iute, A., Fulcher, B.D., Fornito, A., 2019. A practical guide to linking brain-wide gene expression and neuroimaging data. doi:10.1016/j.neuroimage.2019.01.011. 30648605

[bib0010] Bareham C.A., Allanson J., Roberts N., Hutchinson P.J.A., Pickard J.D., Menon D.K., Chennu S. (2018). Longitudinal bedside assessments of brain networks in disorders of consciousness: case reports from the field. Front. Neurol..

[bib0011] Barttfeld P., Uhrig L., Sitt J.D., Sigman M., Jarraya B., Dehaene S. (2015). Signature of consciousness in the dynamics of resting-state brain activity. Proc. Natl. Acad. Sci..

[bib0012] Bastos A.M., Donoghue J.A., Brincat S.L., Mahnke M., Yanar J., Correa J., Waite A.S., Lundqvist M., Roy J., Brown E.N., Miller E.K. (2021). Neural effects of propofol-induced unconsciousness and its reversal using thalamic stimulation. eLife.

[bib0013] Bensaid S., Modolo J., Merlet I., Wendling F., Benquet P. (2019). COALIA: a computational model of human EEG for consciousness research. Front. Syst. Neurosci..

[bib0014] Beppi C., Penner M., Straumann D., Bögli S.Y. (2023). Biomechanical induction of mild brain trauma in larval zebrafish: effects on visual startle reflex habituation. Brain Commun..

[bib0015] Betzel, R.F., Gu, S., Medaglia, J.D., Pasqualetti, F., Bassett, D.S., 2016. Optimally controlling the human connectome: the role of network topology. 10.1038/srep30770 PMC4965758 27468904

[bib0016] Bodart, O., Gosseries, O., Wannez, S., Thibaut, A., Annen, J., Boly, M., Rosanova, M., Casali, A.G., Casarotto, S., Tononi, G., Massimini, M., Laureys, S., 2017. Measures of metabolism and complexity in the brain of patients with disorders of consciousness. doi:10.1016/j.nicl.2017.02.002. PMC5318348 28239544

[bib0017] Bodien Y.G., Katz D.I., Schiff N.D., Giacino J.T. (2022). Behavioral assessment of patients with disorders of consciousness. Semin. Neurol..

[bib0018] Bonhomme V., Staquet C., Montupil J., Defresne A., Kirsch M., Martial C., Vanhaudenhuyse A., Chatelle C., Larroque S.K., Raimondo F., Demertzi A., Bodart O., Laureys S., Gosseries O. (2019). General anesthesia: a probe to explore consciousness. Front. Syst. Neurosci..

[bib0019] Breakspear M. (2017). Dynamic models of large-scale brain activity. Nat. Neurosci..

[bib0020] Burt J., Preller K., Demirtaş M., Ji J.L., Krystal J., Vollenweider F., Anticevic A., Murray J. (2021). Transcriptomics-informed large-scale cortical model captures topography of pharmacological neuroimaging effects of LSD. eLife.

[bib0021] Busch E.L., Huang J., Benz A., Wallenstein T., Lajoie G., Wolf G., Turk-Browne N.B. (2023). Multi-view manifold learning of human brain-state trajectories. Nat. Comput. Sci..

[bib0022] Cabral J., Hugues E., Kringelbach M.L., Deco G. (2012). Modeling the outcome of structural disconnection on resting-state functional connectivity. Neuroimage.

[bib0023] Cabral J., Kringelbach M.L., Deco G. (2017). Functional connectivity dynamically evolves on multiple time-scales over a static structural connectome: models and mechanisms. Neuroimage.

[bib0024] Cabral J., Vidaurre D., Marques P., Magalhães R., Silva Moreira P., Miguel Soares J., Kringelbach M.L. (2017). Cognitive performance in healthy older adults relates to spontaneous switching between states of functional connectivity during rest. Sci. Rep..

[bib0025] Campbell J.M., Huang Z., Zhang J., Wu X., Qin P., Northoff G., Mashour G.A., Hudetz A.G. (2020). Pharmacologically informed machine learning approach for identifying pathological states of unconsciousness via resting-state fMRI. Neuroimage.

[bib0026] Candia-Rivera D., Gosseries O., Annen J., Martial C., Thibaut A., Laureys S., Tallon-Baudry C. (2021). Neural responses to heartbeats detect residual signs of consciousness during resting state in postcomatose patients. J. Neurosci..

[bib0027] Candia-Rivera, D., Raimondo, F., Pérez, P., Naccache, L., Tallon-Baudry, C., & Sitt, J.D. 2023. Conscious processing of global and local auditory irregularities causes differentiated heartbeat-evoked responses. medarXiv. doi:10.1101/2021.10.27.21265539. PMC10651171 37888955

[bib0028] Cao B., Chen Y., Yu R., Chen L., Chen P., Weng Y., Chen Q., Song J., Xie Q., Huang R. (2019). Abnormal dynamic properties of functional connectivity in disorders of consciousness. Neuroimage Clin..

[bib0029] Casali A.G., Gosseries O., Rosanova M., Boly M., Sarasso S., Casali K.R., Casarotto S., Bruno M.A., Laureys S., Tononi G., Massimini M. (2013). A theoretically based index of consciousness independent of sensory processing and behavior. Sci. Transl. Med..

[bib0030] Casarotto S., Comanducci A., Rosanova M., Sarasso S., Fecchio M., Napolitani M., Pigorini A., Casali A.G., Trimarchi P.D., Boly M., Gosseries O., Bodart O., Curto F., Landi C., Mariotti M., Devalle G., Laureys S., Tononi G., Massimini M. (2016). Stratification of unresponsive patients by an independently validated index of brain complexity. Ann. Neurol..

[bib0031] Casey C.P., Tanabe S., Farahbakhsh Z., Parker M., Bo A., White M., Ballweg T., Mcintosh A., Filbey W., Banks M.I., Saalmann Y.B., Pearce R.A., Sanders R.D. (2022). Dynamic causal modelling of auditory surprise during disconnected consciousness: the role of feedback connectivity. Neuroimage.

[bib0032] Castelvecchi D. (2016). Can we open the black box of AI?. Nat. News.

[bib0033] Chennu S., Annen J., Wannez S., Thibaut A., Chatelle C., Cassol H., Martens G.R., Schnakers C., Gosseries O., Menon D., Laureys S. (2017). Brain networks predict metabolism, diagnosis and prognosis at the bedside in disorders of consciousness. Brain.

[bib0034] Ching S., Cimenser A., Purdon P.L., Brown E.N., Kopell N.J. (2010). Thalamocortical model for a propofol-induced α-rhythm associated with loss of consciousness. Proc. Natl. Acad. Sci. USA.

[bib0035] Ching S.N., Purdon P.L., Vijayan S., Kopell N.J., Brown E.N. (2012). A neurophysiological-metabolic model for burst suppression. Proc. Natl. Acad. Sci. USA.

[bib0036] Claassen J., Akbari Y., Alexander S., Bader M.K., Bell K., Bleck T.P., Boly M., Brown J., Chou S.H., Diringer M.N., Edlow B.L., Foreman B., Giacino J.T., Gosseries O., Green T., Greer D.M., Hanley D.F., Hartings J.A., Helbok R., Hemphill J.C., Hinson H.E., Hirsch K., Human T., James M.L., Ko N., Kondziella D., Livesay S., Madden L.K., Mainali S., Mayer S.A., McCredie V., McNett M.M., Meyfroidt G., Monti M.M., Muehlschlegel S., Murthy S., Nyquist P., Olson D.M., Provencio J.J., Rosenthal E., Sampaio Silva G., Sarasso S., Schiff N.D., Sharshar T., Shutter L., Stevens R.D., Vespa P., Videtta W., Wagner A., Ziai W., Whyte J., Zink E., Suarez J.I. (2021). Proceedings of the first curing coma campaign NIH symposium: challenging the future of research for coma and disorders of consciousness. Neurocrit. Care.

[bib0037] Claassen J., Doyle K., Matory A., Couch C., Burger K.M., Velazquez A. (2019). Detection of brain activation in unresponsive patients with acute brain injury. N. Engl. J. Med..

[bib0038] Cofré R., Herzog R., Mediano P.A.M., Piccinini J., Rosas F.E., Perl Y.S., Tagliazucchi E. (2020). Whole-brain models to explore altered states of consciousness from the bottom up. Brain Sci..

[bib0039] Cornblath, E.J., Tang, E., Baum, G.L., Moore, T.M., Roalf, D.R., Gur, R.C., Gur, R.E., Pasqualetti, F., Satterthwaite, T.D., Bassett, D.S., 2018. Sex differences in network controllability as a predictor of executive function in youth. 10.1016/j.neuroimage.2018.11.048 PMC6401302 30508681

[bib0040] Coronel-Oliveros C., Cofré R., Orio P. (2021). Cholinergic neuromodulation of inhibitory interneurons facilitates functional integration in whole-brain models. PLoS Comput. Biol..

[bib0041] Coronel-Oliveros C., Gießing C., Medel V., Cofré R., Orio P. (2023). Whole-brain modeling explains the context-dependent effects of cholinergic neuromodulation. Neuroimage.

[bib0042] D'Angelo E., Jirsa V. (2022). The quest for multiscale brain modeling. Trends Neurosci..

[bib0045] Deco G., Cruzat J., Cabral J., Whybrow P.C., Logothetis N.K., Kringelbach M.L. (2018). Whole-brain multimodal neuroimaging model using serotonin receptor maps explains non-linear functional effects of LSD. Curr. Biol..

[bib0043] Deco G., Cabral J., Saenger V.M., Boly M., Tagliazucchi E., Laufs H., Van Someren E., Jobst B., Stevner A., Kringelbach M.L. (2018). Perturbation of whole-brain dynamics in silico reveals mechanistic differences between brain states. Neuroimage.

[bib0044] Deco G., Cruzat J., Cabral J., Tagliazucchi E., Laufs H., Logothetis N.K., Kringelbach M.L. (2019). Awakening: predicting external stimulation to force transitions between different brain states. Proc. Natl. Acad. Sci..

[bib0046] Deco G., Kringelbach M.L. (2017). Hierarchy of information processing in the brain: a novel “Intrinsic Ignition” framework. Neuron.

[bib0047] Deco G., Kringelbach M.L. (2016). Metastability and coherence: extending the communication through coherence hypothesis using a whole-brain computational perspective. Trends Neurosci..

[bib0048] Deco G., Kringelbach M.L. (2014). Great expectations: using whole-brain computational connectomics for understanding neuropsychiatric disorders. Neuron.

[bib0049] Deco G., Kringelbach M.L., Arnatkeviciute A., Oldham S., Sabaroedin K., Rogasch N.C., Aquino K.M., Fornito A. (2021). Dynamical consequences of regional heterogeneity in the brain's transcriptional landscape. Sci. Adv..

[bib0050] Deco G., Ponce-Alvarez A., Hagmann P., Romani G.L., Mantini D., Corbetta M. (2014). How local excitation-inhibition ratio impacts the whole brain dynamics. J. Neurosci..

[bib0051] Deco G., Ponce-Alvarez A., Mantini D., Romani G.L., Hagmann P., Corbetta M. (2013). Resting-state functional connectivity emerges from structurally and dynamically shaped slow linear fluctuations. J. Neurosci..

[bib0052] Deco G., Tagliazucchi E., Laufs H., Sanjuán A., Kringelbach M.L. (2017). Novel intrinsic ignition method measuring local-global integration characterizes wakefulness and deep sleep. eNeuro.

[bib0053] Demertzi A., Antonopoulos G., Heine L., Voss H.U., Crone J.S., De Los Angeles C., Bahri M.A., Di Perri C., Vanhaudenhuyse A., Charland-Verville V., Kronbichler M., Trinka E., Phillips C., Gomez F., Tshibanda L., Soddu A., Schiff N.D., Whitfield-Gabrieli S., Laureys S. (2015). Intrinsic functional connectivity differentiates minimally conscious from unresponsive patients. Brain.

[bib0054] Demertzi A., Tagliazucchi E., Dehaene S., Deco G., Barttfeld P., Raimondo F., Martial C., Fernández-Espejo D., Rohaut B., Voss H.U., Schiff N.D., Owen A.M., Laureys S., Naccache L., Sitt J.D. (2019). Human consciousness is supported by dynamic complex patterns of brain signal coordination. Sci. Adv..

[bib0055] Demirtaş M., Burt J.B., Helmer M., Ji J.L., Adkinson B.D., Glasser M.F., Van Essen D.C., Sotiropoulos S.N., Anticevic A., Murray J.D. (2019). Hierarchical heterogeneity across human cortex shapes large-scale neural dynamics. Neuron.

[bib0056] Donoghue, J.A., Bastos, A.M., Yanar, J., Kornblith, S., Mahnke, M., Brown, E.N., Miller, E.K., 2019. Neural signatures of loss of consciousness and its recovery by thalamic stimulation. bioRxiv. 10.1101/806687

[bib0057] Edlow B.L., Claassen J., Schiff N.D., Greer D.M. (2020). Recovery from disorders of consciousness: mechanisms, prognosis and emerging therapies. Nat. Rev. Neurol..

[bib0058] Einevoll G.T., Destexhe A., Diesmann M., Grün S., Jirsa V., de Kamps M., Migliore M., Ness T.V., Plesser H.E., Schürmann F. (2019). The scientific case for brain simulations. Neuron.

[bib0059] Engemann D.A., Raimondo F., King J.R.M., Rohaut B., Louppe G., Dé F., Faugeras R., Annen J., Cassol H., Gosseries O., Fernandez-Slezak D., Laureys S., Naccache L., Dehaene S., Sitt J.D., Sitt J. (2018). Robust EEG-based cross-site and cross-protocol classification of states of consciousness. Brain.

[bib0060] Erol T., Mendi A.F., Doğan D. (2020). Proceedings of the 4th International Symposium on Multidisciplinary Studies and Innovative Technologies (ISMSIT).

[bib0061] Estraneo A., Fiorenza S., Magliacano A., Formisano R., Mattia D., Grippo A., Romoli A.M., Angelakis E., Cassol H., Thibaut A., Gosseries O., Lamberti G., Noé E., Bagnato S., Edlow B.L., Chatelle C., Lejeune N., Veeramuthu V., Bartolo M., Toppi J., Zasler N., Schnakers C., Trojano L. (2020). Multicenter prospective study on predictors of short-term outcome in disorders of consciousness. Neurology.

[bib176] Farinha M., Amado C., Morgado P., Cabral J. (2022). Increased excursions to functional networks in schizophrenia in the absence of task. Front. Neurosci..

[bib0062] Favaretto C., Allegra M., Deco G., Metcalf N.V., Griffis J.C., Shulman G.L., Corbetta M. (2022). Subcortical-cortical dynamical states of the human brain and their breakdown in stroke. Nat. Commun..

[bib0063] Friedman E.B., Sun Y., Moore J.T., Hung H.T., Meng Q.C., Perera P., Joiner W.J., Thomas S.A., Eckenhoff R.G., Sehgal A., Kelz M.B. (2010). A conserved behavioral state barrier impedes transitions between anesthetic-induced unconsciousness and wakefulness: evidence for neural inertia. PLoS ONE.

[bib0064] Friston K.J., Harrison L., Penny W. (2003). Dynamic causal modelling. Neuroimage.

[bib0065] Friston K.J., Preller K.H., Mathys C., Cagnan H., Heinzle J., Razi A., Zeidman P. (2019). Dynamic causal modelling revisited. Neuroimage.

[bib0066] Giacino J. (2004). The vegetative and minimally conscious states: consensus-based criteria for establishing diagnosis and prognosis. NeuroRehabilitation.

[bib0067] Giacino J.T., Ashwal S., Childs N., Cranford R., Jennett B., Katz D.I., Kelly J.P., Rosenberg J.H., Whyte J., Zafonte R.D., Zasler N.D. (2002). The minimally conscious state: definition and diagnostic criteria. Neurology.

[bib0068] Gilson M., Zamora-López G., Pallarés V., Adhikari M.H., Senden M., Campo A.T., Mantini D., Corbetta M., Deco G., Insabato A. (2019). Model-based whole-brain effective connectivity to study distributed cognition in health and disease. Netw. Neurosci..

[bib0069] Goldman, J.S., Kusch, L., Yalçinkaya, B.H., Depannemaecker, D., Nghiem, T.A.E., Jirsa, V., Destexhe, A., 2021. A comprehensive neural simulation of slow-wave sleep and highly responsive wakefulness dynamics. doi:10.1101/2021.08.31.458365. PMC9880280 36714530

[bib0070] Golkowski D., Merz K., Mlynarcik C., Kiel T., Schorr B., Lopez-Rolon A., Lukas M., Jordan D., Bender A., Ilg R. (2017). Simultaneous EEG–PET–fMRI measurements in disorders of consciousness: an exploratory study on diagnosis and prognosis. J. Neurol..

[bib0071] Golkowski D., Willnecker R., Rösler J., Ranft A., Schneider G., Jordan D., Ilg R. (2021). Dynamic patterns of global brain communication differentiate conscious from unconscious patients after severe brain injury. Front. Syst. Neurosci..

[bib0072] Goulas A., Changeux J.P., Wagstyl K., Amunts K., Palomero-Gallagher N., Hilgetag C.C. (2021). The natural axis of transmitter receptor distribution in the human cerebral cortex. Proc. Natl. Acad. Sci. USA.

[bib0073] Gu S., Pasqualetti F., Cieslak M., Telesford Q.K., Yu A.B., Kahn A.E., Medaglia J.D., Vettel J.M., Miller M.B., Grafton S.T., Bassett D.S. (2015). Controllability of structural brain networks. Nat. Commun..

[bib0074] Gu Z., Jamison K., Sabuncu M., Kuceyeski A. (2022). Personalized visual encoding model construction with small data. Commun. Biol..

[bib0075] Gutierrez-Barragan D., Singh N.A., Alvino F.G., Coletta L., Rocchi F., De Guzman E., Galbusera A., Uboldi M., Panzeri S., Gozzi A. (2021). Unique spatiotemporal fMRI dynamics in the awake mouse brain. Curr. Biol. CB.

[bib0076] Hahn G., Zamora-López G., Uhrig L., Tagliazucchi E., Laufs H., Mantini D., Kringelbach M., Jarraya B., Deco G. (2020). Signature of consciousness in brain-wide synchronization patterns of monkey and human fMRI signals. Neuroimage.

[bib0077] Hansen J.Y., Shafiei G., Markello R.D., Cox S., Smart K., Aumont É., Servaes S., Scala S., Wainstein G., Bezgin G., Funck T., Schmitz W., Bédard M., Spreng R.N., Soucy J., Guimond S. (2022). Mapping neurotransmitter systems to the structural and functional organization of the human neocortex. Nat. Neurosci..

[bib0078] Hawrylycz M.J., Lein E.S., Guillozet-Bongaarts A.L., Shen E.H., Ng L., Miller J.A., Van De Lagemaat L.N., Smith K.A., Ebbert A., Riley Z.L., Abajian C., Beckmann C.F., Bernard A., Bertagnolli D., Boe A.F., Cartagena P.M., Mallar Chakravarty M., Chapin M., Chong J., Dalley R.A., Daly B.D., Dang C., Datta S., Dee N., Dolbeare T.A., Faber V., Feng D., Fowler D.R., Goldy J., Gregor B.W., Haradon Z., Haynor D.R., Hohmann J.G., Horvath S., Howard R.E., Jeromin A., Jochim J.M., Kinnunen M., Lau C., Lazarz E.T., Lee C., Lemon T.A., Li L., Li Y., Morris J.A., Overly C.C., Parker P.D., Parry S.E., Reding M., Royall J.J., Schulkin J., Sequeira P.A., Slaughterbeck C.R., Smith S.C., Sodt A.J., Sunkin S.M., Swanson B.E., Vawter M.P., Williams D., Wohnoutka P., Ronald Zielke H., Geschwind D.H., Hof P.R., Smith S.M., Koch C., Grant S.G.N., Jones A.R. (2012). An anatomically comprehensive atlas of the adult human brain transcriptome. Nature.

[bib0079] Heinrichs B., Eickhoff S.B. (2020). Your evidence? Machine learning algorithms for medical diagnosis and prediction. Hum. Brain Mapp..

[bib0080] Hellyer P.J., Jachs B., Clopath C., Leech R. (2016). Local inhibitory plasticity tunes macroscopic brain dynamics and allows the emergence of functional brain networks. Neuroimage.

[bib0081] Hermann B., Stender J., Habert M.O., Kas A., Denis-Valente M., Raimondo F., Pérez P., Rohaut B., Sitt J.D., Naccache L. (2021). Multimodal FDG-PET and EEG assessment improves diagnosis and prognostication of disorders of consciousness. Neuroimage Clin..

[bib0082] Huang Z., Zhang J., Wu J., Mashour G.A., Hudetz A.G. (2020). Temporal circuit of macroscale dynamic brain activity supports human consciousness. Sci. Adv..

[bib0083] Huntley J.D., Fleming S.M., Mograbi D.C., Bor D., Naci L., Owen A.M., Howard R. (2021). Understanding Alzheimer's disease as a disorder of consciousness. Alzheimer's Dement..

[bib0084] Hutchison R.M., Hutchison M., Manning K.Y., Menon R.S., Everling S. (2014). Isoflurane induces dose-dependent alterations in the cortical connectivity profiles and dynamic properties of the brain's functional architecture. Hum. Brain Mapp..

[bib0085] Ipiña I.P., Kehoe P.D., Kringelbach M., Laufs H., Ibañez A., Deco G., Perl Y.S., Tagliazucchi E. (2020). Modeling regional changes in dynamic stability during sleep and wakefulness. Neuroimage.

[bib0086] Jobst B.M., Hindriks R., Laufs H., Tagliazucchi E., Hahn G., Ponce-Alvarez A., Stevner A.B.A., Kringelbach M.L., Deco G. (2017). Increased stability and breakdown of brain effective connectivity during slow-wave sleep: mechanistic insights from whole-brain computational modelling. Sci. Rep..

[bib0087] Kandeepan S., Rudas J., Gomez F., Stojanoski B., Valluri S., Owen A.M., Naci L., Nichols E.S., Soddu A. (2020). Modeling an auditory stimulated brain under altered states of consciousness using the generalized ising model. Neuroimage.

[bib0088] Khosla M., Jamison K., Kuceyeski A., Sabuncu M.R. (2019). Proceedings of the 10th International Workshop, Machine Learning in Medical Imaging.

[bib0089] Kim J.Z., Soffer J.M., Kahn A.E., Vettel J.M., Pasqualetti F., Bassett D.S. (2018). Role of graph architecture in controlling dynamical networks with applications to neural systems. Nat. Phys..

[bib0090] King J.R., Sitt J.D., Faugeras F., Rohaut B., El Karoui I., Cohen L., Dehaene S. (2013). Information sharing in the brain indexes consciousness in noncommunicative patients. Curr. Biol..

[bib0091] Kringelbach M.L., Cruzat J., Cabral J., Knudsen G.M., Carhart-Harris R., Whybrow P.C., Logothetis N.K., Deco G. (2020). Dynamic coupling of whole-brain neuronal and neurotransmitter systems. Proc. Natl. Acad. Sci. USA.

[bib0092] Kringelbach M.L., Deco G. (2020). Brain states and transitions: insights from computational neuroscience. Cell Rep..

[bib0093] Kuceyeski A., Shah S., Dyke J.P., Bickel S., Abdelnour F., Schiff N.D., Voss H.U., Raj A. (2016). The application of a mathematical model linking structural and functional connectomes in severe brain injury. Neuroimage Clin..

[bib0094] Kuizenga M.H., Colin P.J., Reyntjens K.M.E.M., Touw D.J., Nalbat H., Knotnerus F.H., Vereecke H.E.M., Struys M.M.R.F. (2018). Test of neural inertia in humans during general anaesthesia. Br. J. Anaesth..

[bib0095] Kurtin D.L., Giunchiglia V., Vohryzek J., Cabral J., Skeldon A.C., Violante I.R. (2023). Moving from phenomenological to predictive modelling: progress and pitfalls of modelling brain stimulation in-silico. Neuroimage.

[bib0096] Lee M., Sanz L.R., Barra A., Wolff A., Nieminen J.O., Boly M., Lee S.W. (2022). Quantifying arousal and awareness in altered states of consciousness using interpretable deep learning. Nat. Commun..

[bib0097] López-González A., Panda R., Ponce-Alvarez A., Zamora-López G., Escrichs A., Martial C., Thibaut A., Gosseries O., Kringelbach M.L., Annen J., Laureys S., Deco G. (2021). Loss of consciousness reduces the stability of brain hubs and the heterogeneity of brain dynamics. Commun. Biol..

[bib0098] Lord L.D., Expert P., Atasoy S., Roseman L., Rapuano K., Lambiotte R., Nutt D.J., Deco G., Carhart-Harris R.L., Kringelbach M.L., Cabral J. (2019). Dynamical exploration of the repertoire of brain networks at rest is modulated by psilocybin. Neuroimage.

[bib0099] Luppi A.I., Cabral J., Cofre R., Destexhe A., Deco G., Kringelbach M.L. (2022). Dynamical models to evaluate structure–function relationships in network neuroscience. Nat. Rev. Neurosci..

[bib0100] Luppi A.I., Cain J., Spindler L.R.B., Górska U.J., Toker D., Hudson A.E., Brown E.N., Diringer M.N., Stevens R.D., Massimini M., Monti M.M., Stamatakis E.A., Boly M., Curing Coma Campaign (2021). Mechanisms underlying disorders of consciousness: bridging gaps to move toward an integrated translational science. Neurocrit. Care.

[bib0101] Luppi A.I., Craig M.M., Coppola P., Peattie A.R.D., Finoia P., Williams G.B., Allanson J., Pickard J.D., Menon D.K., Stamatakis E.A. (2021). Preserved fractal character of structural brain networks is associated with covert consciousness after severe brain injury. Neuroimage Clin..

[bib0102] Luppi A.I., Craig M.M., Pappas I., Finoia P., Williams G.B., Allanson J., Pickard J.D., Owen A.M., Naci L., Menon D.K., Stamatakis E.A. (2019). Consciousness-specific dynamic interactions of brain integration and functional diversity. Nat. Commun..

[bib0103] Luppi A.I., Mediano P.A.M., Rosas F.E., Allanson J., Pickard J.D., Williams G.B., Craig M.M., Finoia P., Peattie A.R.D., Coppola P., Menon D.K., Bor D., Stamatakis E.A. (2023). Reduced emergent character of neural dynamics in patients with a disrupted connectome. Neuroimage.

[bib0104] Luppi A.I., Mediano P.A.M., Rosas F.E., Allanson J., Pickard J.D., Williams G.B., Craig M.M., Finoia P., Peattie A.R.D., Coppola P., Owen A.M., Naci L., Menon D.K., Bor D., Stamatakis E.A. (2022). Whole-brain modelling identifies distinct but convergent paths to unconsciousness in anaesthesia and disorders of consciousness. Commun. Biol..

[bib0105] Luppi A.I., Spindler L.R.B., Menon D.K., Stamatakis E.A. (2021). The inert brain: explaining neural inertia as post-anaesthetic sleep inertia. Front. Neurosci..

[bib0106] Lutkenhoff E.S., Johnson M.A., Casarotto S., Massimini M., Monti M.M. (2020). Subcortical atrophy correlates with the perturbational complexity index in patients with disorders of consciousness. Brain Stimul..

[bib0107] Lynn C.W., Bassett D.S. (2019). The physics of brain network structure, function and control. Nat. Rev. Phys..

[bib0108] Maas A.I.R., Ercole A., De Keyser V., Menon D.K., Steyerberg E.W. (2022). Opportunities and challenges in high-quality contemporary data collection in traumatic brain injury: the CENTER-TBI experience. Neurocrit. Care.

[bib0109] Maas A.I.R., Menon D.K., Steyerberg E.W., Citerio G., Lecky F., Manley G., Hill S., Legrand V., Sorgner A. (2014). Collaborative European NeuroTrauma effectiveness research in traumatic brain injury (CENTER-TBI): a prospective longitudinal observational study. Neurosurgery.

[bib0110] Markello R.D., Arnatkevičiūtė A., Poline J.B., Fulcher B.D., Fornito A., Misic B. (2021). Standardizing workflows in imaging transcriptomics with the Abagen toolbox. eLife.

[bib0111] Markello R.D., Hansen J.Y., Liu Z.Q., Bazinet V., Shafiei G., Suárez L.E., Blostein N., Seidlitz J., Baillet S., Satterthwaite T.D., Chakravarty M.M., Raznahan A., Misic B. (2022). neuromaps: structural and functional interpretation of brain maps. Nat. Methods.

[bib0112] Medaglia J.D., Pasqualetti F., Hamilton R.H., Thompson-Schill S.L., Bassett D.S. (2017). Brain and cognitive reserve: translation via network control theory. Neurosci. Biobehav. Rev..

[bib0113] Michael Milham A.P., Ai L., Koo B., Zhou Y., Margulies D.S., Schroeder Correspondence C.E., Milham M.P., Xu T., line Amiez C., Balezeau F., Baxter M.G., Blezer E.L., Brochier T., Chen A., Croxson P.L., Damatac C.G., Dehaene S., Everling S., Fair D.A., Fleysher L., Freiwald W., Froudist-Walsh S., Griffiths T.D., Guedj C., Hadj-Bouziane F., Ben Hamed S., Harel N., Hiba B., Jarraya B., Jung B., Kastner S., Christiaan Klink P., Chai Kwok S., Laland K.N., Leopold D.A., Mok K., Morrison J.H., Nacef J., Nagy J., Ortiz Rios M., Petkov C.I., Pinsk M., Poirier C., Procyk E., Rajimehr R. (2018). An open resource for non-human primate imaging. Neuron.

[bib0114] Monti M.M., Vanhaudenhuyse A., Coleman M.R., Boly M., Pickard J.D., Tshibanda L., Owen A.M., Laureys S. (2010). Willful modulation of brain activity in disorders of consciousness. N. Engl. J. Med..

[bib0115] Müller E.J., Munn B.R., Shine J.M. (2020). Diffuse neural coupling mediates complex network dynamics through the formation of quasi-critical brain states. Nat. Commun..

[bib0116] Naci L., Sinai L., Owen A.M. (2017). Detecting and interpreting conscious experiences in behaviorally non-responsive patients. Neuroimage.

[bib0117] Noormandi A., Shahrokhi M., Khalili H. (2017). Potential benefits of zolpidem in disorders of consciousness. Expert Rev. Clin. Pharmacol..

[bib0118] Olafson, E., Russello, G., Jamison, K.W., Liu, H., Wang, D., Bruss, J.E., Boes, A.D., Kuceyeski, A., 2022. Increased prevalence of a frontoparietal brain state is associated with better motor recovery after stroke affecting dominant-hand corticospinal tract. doi:10.1101/2022.02.10.479962.

[bib0119] Olafson E.R., Jamison K.W., Sweeney E.M., Liu H., Wang D., Bruss J.E., Boes A.D., Kuceyeski A. (2021). Functional connectome reorganization relates to post-stroke motor recovery and structural and functional disconnection. Neuroimage.

[bib0120] Olsen A.S., Lykkebo-Valløe A., Ozenne B., Madsen M.K., Stenbæk D.S., Armand S., Fisher P.M. (2022). Psilocybin modulation of time-varying functional connectivity is associated with plasma psilocin and subjective effects. Neuroimage.

[bib0121] Orlhac F., Eertink J.J., Cottereau A.S., Zijlstra J.M., Thieblemont C., Meignan M., Boellaard R., Buvat I. (2022). A Guide to ComBat Harmonization of Imaging Biomarkers in Multicenter Studies. J. Nucl. Med..

[bib0122] Ou J., Xie L., Jin C., Li X., Zhu D., Jiang R., Chen Y., Zhang J., Li L., Liu T. (2015). Characterizing and differentiating brain state dynamics via Hidden Markov models. Brain Topogr..

[bib0123] Owen A.M., Coleman M.R., Boly M., Davis M.H., Laureys S., Pickard J.D. (2006). Detecting awareness in the vegetative state. Science.

[bib0124] Panda R., Thibaut A., Lopez-Gonzalez A., Escrichs A., Bahri M.A., Hillebrand A., Deco G., Laureys S., Gosseries O., Annen J., Tewarie P. (2022). Disruption in structural-functional network repertoire and time-resolved subcortical fronto-temporoparietal connectivity in disorders of consciousness. eLife.

[bib0125] Pasqualetti F., Gu S., Bassett D.S. (2019). RE: warnings and caveats in brain controllability. Neuroimage.

[bib0126] Pathak A., Roy D., Banerjee A. (2022). Whole-brain network models: from physics to bedside. Front. Comput. Neurosci..

[bib0127] Perl Y.S., Bocaccio H., Pérez-Ipiña I., Zamberlán F., Piccinini J., Laufs H., Kringelbach M., Deco G., Tagliazucchi E. (2020). Generative embeddings of brain collective dynamics using variational autoencoders. Phys. Rev. Lett..

[bib0128] Pomponio R., Erus G., Habes M., Doshi J., Srinivasan D., Mamourian E., Bashyam V., Nasrallah I.M., Satterthwaite T.D., Fan Y., Launer L.J., Masters C.L., Maruff P., Zhuo C., Völzke H., Johnson S.C., Fripp J., Koutsouleris N., Wolf D.H., Gur Raquel, Gur Ruben, Morris J., Albert M.S., Grabe H.J., Resnick S.M., Bryan R.N., Wolk D.A., Shinohara R.T., Shou H., Davatzikos C. (2020). Harmonization of large MRI datasets for the analysis of brain imaging patterns throughout the lifespan. Neuroimage.

[bib0129] Preller K.H., Razi A., Zeidman P., Stämpfli P., Friston K.J., Vollenweider F.X. (2019). Effective connectivity changes in LSD-induced altered states of consciousness in humans. Proc. Natl. Acad. Sci. USA.

[bib0130] Preti M.G., Bolton T.A., Van De Ville D. (2017). The dynamic functional connectome: state-of-the-art and perspectives. Neuroimage.

[bib0131] Proekt A., Hudson A.E. (2018). A stochastic basis for neural inertia in emergence from general anaesthesia. Br. J. Anaesth..

[bib0132] Qin P., Wu Xuehai, Duncan N.W., Bao W., Tang W., Zhang Z., Hu J., Jin Y., Wu Xing, Gao L., Lu L., Guan Y., Lane T., Huang Z., Bodien Y.G., Giacino J.T., Mao Y., Northoff G. (2015). GABAA receptor deficits predict recovery in patients with disorders of consciousness: a preliminary multimodal [(11) C]Flumazenil PET and fMRI study. Hum. Brain Mapp..

[bib0133] Raimondo F., Rohaut B., Demertzi A., Valente M., Engemann D.A., Salti M., Sitt J.D. (2017). Brain–heart interactions reveal consciousness in noncommunicating patients. Ann. Neurol..

[bib0134] Ramezanian-Panahi M., Abrevaya G., Gagnon-Audet J.C., Voleti V., Rish I., Dumas G. (2022). Generative models of brain dynamics. Front. Artif. Intell..

[bib0135] Redinbaugh M.J., Phillips J.M., Kambi N.A., Mohanta S., Andryk S., Dooley G.L., Afrasiabi M., Raz A., Saalmann Y.B. (2020). Thalamus modulates consciousness via layer-specific control of cortex. Neuron.

[bib0136] Riganello F., Karl Larroque S., Ali Bahri M., Heine L., Martial C., Carrière M., Charland-Verville V., Aubinet C., Vanhaudenhuyse A., Chatelle C., Laureys S., Di Perri C. (2018). A heartbeat away from consciousness: heart rate variability entropy can discriminate disorders of consciousness and is correlated with resting-state fmri brain connectivity of the central autonomic network. Front. Neurol..

[bib0137] Rocha R.P., Koçillari L., Suweis S., De Filippo De Grazia M., de Schotten M.T., Zorzi M., Corbetta M. (2022). Recovery of neural dynamics criticality in personalized whole-brain models of stroke. Nat. Commun..

[bib0138] Roland P.E., Rosanova M., Jennifer van Albada S., Goldman J.S., di Volo M., Tort-Colet N., Susin E., Bouté J., Dali M., Carlu M., Nghiem T.A., Górski T., Destexhe A. (2019). Bridging single neuron dynamics to global brain states. Front. Syst. Neurosci..

[bib0139] Rosanova M., Fecchio M., Casarotto S., Sarasso S., Casali A.G., Pigorini A., Comanducci A., Seregni F., Devalle G., Citerio G., Bodart O., Boly M., Gosseries O., Laureys S., Massimini M. (2018). Sleep-like cortical OFF-periods disrupt causality and complexity in the brain of unresponsive wakefulness syndrome patients. Nat. Commun..

[bib0140] Sala A., Annen J., Thibaut A., Laureys S. (2021). Disturbance of brain glucose metabolism in disorders of consciousness: a meta-analysis. J. Nucl. Med..

[bib0141] Sanz Perl Y., Pallavicini C., Pérez Ipiña I., Demertzi A., Bonhomme V., Martial C., Panda R., Annen J., Ibañez A., Kringelbach M., Deco G., Laufs H., Sitt J., Laureys S., Tagliazucchi E. (2021). Perturbations in dynamical models of whole-brain activity dissociate between the level and stability of consciousness. PLoS Comput. Biol..

[bib0142] Sanz Perl Y., Pallavicini C., Perez Ipiña I., Kringelbach M., Deco G., Laufs H., Tagliazucchi E. (2020). Data augmentation based on dynamical systems for the classification of brain states. Chaos Solitons Fractals.

[bib0143] Sarasso S., Casali A.G., Casarotto S., Rosanova M., Sinigaglia C., Massimini M. (2021). Consciousness and complexity: a consilience of evidence. Neurosci. Conscious..

[bib0144] Schnakers C., Vanhaudenhuyse A., Giacino J., Ventura M., Boly M., Majerus S., Moonen G., Laureys S. (2009). Diagnostic accuracy of the vegetative and minimally conscious state: clinical consensus versus standardized neurobehavioral assessment. BMC Neurol..

[bib0145] Schnakers M.M. (2017). Disorders of consciousness after severe brain injury: therapeutic options. Curr. Opin. Neurol..

[bib0146] Shine J.M., Müller E.J., Munn B., Cabral J., Moran R.J., Breakspear M. (2021). Computational models link cellular mechanisms of neuromodulation to large-scale neural dynamics. Nat. Neurosci..

[bib0147] Singleton S.P., Luppi A.I., Carhart-Harris R.L., Cruzat J., Roseman L., Nutt D.J., Deco G., Kringelbach M.L., Stamatakis E.A., Kuceyeski A. (2022). Receptor-informed network control theory links LSD and psilocybin to a flattening of the brain's control energy landscape. Nat. Commun..

[bib0148] Song M., Yang Y., He J., Yang Z., Yu S., Xie Q., Xia X., Dang Y., Zhang Q., Wu X., Cui Y., Hou B., Yu R., Xu R., Jiang T. (2018). Prognostication of chronic disorders of consciousness using brain functional networks and clinical characteristics. eLife.

[bib0149] Spindler L.R.B., Luppi A.I., Adapa R.M., Craig M.M., Coppola P., Peattie A.R.D., Manktelow A.E., Finoia P., Sahakian B.J., Williams G.B., Allanson J., Pickard J.D., Menon D.K., Stamatakis E.A. (2021). Dopaminergic brainstem disconnection is common to pharmacological and pathological consciousness perturbation. Proc. Natl. Acad. Sci. USA.

[bib0150] Stefan S., Schorr B., Lopez-Rolon A., Kolassa I.T., Shock J.P., Rosenfelder M., Heck S., Bender A. (2018). Consciousness indexing and outcome prediction with resting-state EEG in severe disorders of consciousness. Brain Topogr..

[bib0151] Stramaglia S., Pellicoro M., Angelini L., Amico E., Aerts H., Cortés J.M., Marinazzo D. (2017). Ising model with conserved magnetization on the human connectome: implications on the relation structure-function in wakefulness and anesthesia. Chaos Interdiscip. J. Nonlinear Sci..

[bib0152] Sitt J.D., King J.R., El Karoui I., Rohaut B., Faugeras F., Gramfort A., Naccache L. (2014). Large scale screening of neural signatures of consciousness in patients in a vegetative or minimally conscious state. Brain.

[bib0153] Stevner A.B.A., Vidaurre D., Cabral J., Rapuano K., Nielsen S.F.V., Tagliazucchi E., Laufs H., Vuust P., Deco G., Woolrich M.W., Van Someren E., Kringelbach M.L. (2019). Discovery of key whole-brain transitions and dynamics during human wakefulness and non-REM sleep. Nat. Commun..

[bib0154] Stoliker D., Novelli L., Vollenweider F.X., Egan G.F., Preller K.H., Razi A. (2022). Effective connectivity of functionally anticorrelated networks under lysergic acid diethylamide. Biol. Psychiatry.

[bib0156] Suweis S., Tu C., Rocha R.P., Zampieri S., Zorzi M., Corbetta M. (2019). Brain controllability: not a slam dunk yet. Neuroimage.

[bib0157] Tang E., Giusti C., Baum G.L., Gu S., Pollock E., Kahn A.E., Roalf D.R., Moore T.M., Ruparel K., Gur R.C., Gur R.E., Satterthwaite T.D., Bassett D.S. (2017). Developmental increases in white matter network controllability support a growing diversity of brain dynamics. Nat. Commun..

[bib0158] Tang E., Ju H., Baum G.L., Roalf D.R., Satterthwaite T.D., Pasqualetti F., Bassett D.S. (2020). Control of brain network dynamics across diverse scales of space and time. Phys. Rev. E.

[bib0159] Tasserie J., Uhrig L., Sitt J.D., Manasova D., Dupont M., Dehaene S., Jarraya B. (2022). Deep brain stimulation of the thalamus restores signatures of consciousness in a nonhuman primate model. Sci. Adv..

[bib0160] Tu C., Rocha R.P., Corbetta M., Zampieri S., Zorzi M., Suweis S. (2018). Warnings and caveats in brain controllability. Neuroimage.

[bib0161] Uddin L.Q. (2021). Cognitive and behavioural flexibility: neural mechanisms and clinical considerations. Nat. Rev. Neurosci..

[bib173] Varoquaux G. (2018). Cross-validation failure: Small sample sizes lead to large error bars. Neuroimage.

[bib0162] Váša F., Mišić B. (2022). Null models in network neuroscience. Nat. Rev. Neurosci..

[bib0163] Váša F., Shanahan M., Hellyer P.J., Scott G., Cabral J., Leech R. (2015). Effects of lesions on synchrony and metastability in cortical networks. Neuroimage.

[bib0164] Vidaurre D., Quinn A.J., Baker A.P., Dupret D., Tejero-Cantero A., Woolrich M.W. (2016). Spectrally resolved fast transient brain states in electrophysiological data. Neuroimage.

[bib0165] Vidaurre D., Smith S.M., Woolrich M.W. (2017). Brain network dynamics are hierarchically organized in time. Proc. Natl. Acad. Sci. USA.

[bib0166] Vohryzek J., Cabral J., Castaldo F., Sanz-Perl Y., Lord L.D., Fernandes H., Litvak V., Kringelbach M.L., Deco G. (2022). Dynamic sensitivity analysis: defining personalised strategies to drive brain state transitions via whole brain modelling. Comput. Struct. Biotechnol. J..

[bib0167] Vohryzek J., Deco G., Cessac B., Kringelbach M.L., Cabral J. (2020). Ghost attractors in spontaneous brain activity: recurrent excursions into functionally-relevant BOLD phase-locking states. Front. Syst. Neurosci..

[bib0168] Wielek T., Lechinger J., Wislowska M., Blume C., Ott P., Wegenkittl S., del Giudice R., Heib D.P.J., Mayer H.A., Laureys S., Pichler G., Schabus M. (2018). Sleep in patients with disorders of consciousness characterized by means of machine learning. PLoS ONE.

[bib174] Zarghami T.S., Friston K.J. (2020). Dynamic effective connectivity. Neuroimage.

[bib0169] Zarkali A., McColgan P., Ryten M., Reynolds R., Leyland L.A., Lees A.J., Rees G., Weil R.S. (2020). Differences in network controllability and regional gene expression underlie hallucinations in Parkinson's disease. Brain.

[bib0170] Zheng Z.S., Reggente N., Lutkenhoff E., Owen A.M., Monti M.M. (2017). Disentangling disorders of consciousness: insights from diffusion tensor imaging and machine learning. Hum. Brain Mapp..

[bib0171] Zilles K., Palomero-Gallagher N. (2017). Multiple transmitter receptors in regions and layers of the human cerebral cortex. Front. Neuroanat..

